# E3 ubiquitin ligases: structural diversity, dysregulation in disease, and their emerging role in targeted therapeutic strategies

**DOI:** 10.3389/fmolb.2026.1745654

**Published:** 2026-03-10

**Authors:** Srineevas Sriram, Prahalad Krishnakumar, C. Sudandiradoss

**Affiliations:** Department of Biotechnology, School of Bio Sciences and Technology, Vellore Institute of Technology, Vellore, Tamil Nadu, India

**Keywords:** AI, CAR t cell, E3 ligases, PROTACs, proteostasis, therapeutics, ubiquitination

## Abstract

The Ubiquitin-Proteasome System (UPS) is a key mechanism of cellular homeostasis. A central part of this mechanism is E3 ubiquitin ligases, which selectively direct proteins to be ubiquitinated for degradation via the UPS. In this review we give an integrated overview of the classification, structural and the functional characteristics of the main families of E3 ligases, i.e., RING, HECT, RBR and RCR E3 ligases, as well as non-canonical ligase families. Furthermore, we describe how these ligases contribute to several important biological processes like proteostasis, DNA-repair, cell-cycle control, immune-regulation and neurodegeneration. Here we present examples of diseases that occur due to abnormal functioning of E3 ligases (e.g., cancers, neurodegenerative diseases and immune dysfunctions). Finally, the review also covers emerging therapeutic strategies based on E3 ligases with an emphasis on proteolysis-targeting chimeras (PROTACs) and the use of E3 ligase-modulatory approaches to improve CAR-T-cell-based immunotherapies. Recent developments in artificial intelligence and machine learning have already transformed E3-ligase research through the possibility of high-throughput ligand screening, structure-function prediction and rational design of degraders. Our review aims to integrate our knowledge of E3 ligases and show how converging biochemistry, immunotherapy and AI-driven research can lead to novel precision strategies for targeted protein degradation.

## Graphical Abstract

**Figure:**
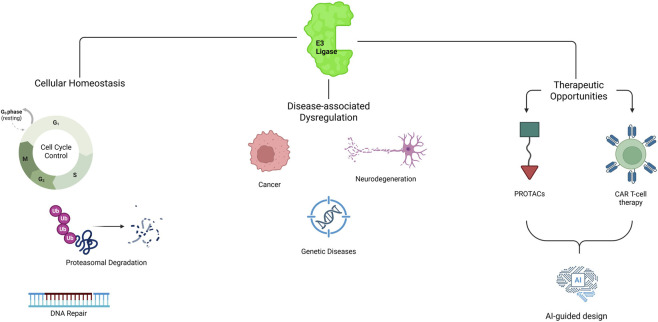
E3 ubiquitin ligases as central regulatory hubs in physiology, disease, and therapeutic innovation. E3 ligases coordinate cellular homeostasis by regulating cell cycle progression, proteostasis, and DNA repair. Dysregulation of E3-mediated ubiquitin signaling contributes to diverse disease states, including cancer, neurodegeneration, and genetic disorders. Advances in therapeutic exploitation of E3 ligases have enabled targeted protein degradation strategies such as PROTACs, emerging immunotherapeutic approaches including CAR-T cell therapy, and AI-guided design frameworks that accelerate discovery and optimization of E3-based interventions. Created in BioRender. Krishnakumar, P. (2026) https://BioRender.com/d19nlqd.

## 1 Introduction

Cellular proteostasis is maintained through continuous cell turnover by two pathways: the ubiquitin proteasome system (UPS) and autophagy (Dikic, 2017). While autophagy is a bulk degradation mechanism for aggregates and organelles, the UPS eliminates short-lived regulatory, misfolded, and damaged proteins by tagging them with ubiquitin through the E3 ligases (Finley, 2009). Dysregulation of either pathway results in upregulation of the other, as evidenced by aggresome formation as a cytoprotective mechanism, implying compensatory cross-talk between the two pathways (Finley, 2009; Wang et al., 2013). However, chronic inhibition of autophagy impairs UPS function, in which case, the oxidative stress builds up due to sequestration of proteins, resulting in overwhelming of cytoprotective mechanisms and apoptosis, driving the pathogenesis of proteinopathies, myopathies, and oncogenic development (Dikic, 2017; Wang et al., 2013).

Specificity is achieved by E3 ubiquitin ligases through selection of substrates and determination of the outcome of ubiquitination to regulate the stability and signaling of proteins and ultimately the fate of cells (Berndsen and Wolberger, 2014; Swatek and Komander, 2016). By virtue of their ability to regulate selective protein turnover and nondegradative ubiquitin signaling, E3 ligases are central regulatory points where proteostasis interfaces with other aspects of cellular regulation. Beyond their role in protein quality control, E3 ligases have also become increasingly relevant in developmental processes and diseases, as illustrated by the role of the cereblon E3 ligase complex in thalidomide sensitive tissues and its potential impact on therapeutic outcomes depending on the context (Fraga et al., 2025).

With an increased understanding of fundamental ubiquitin biology, new therapeutic platforms have emerged that harness the ability of E3 ligases. Molecular Glue molecules and proteolysis-targeting chimeras (PROTACs) target endogenous ubiquitination systems to facilitate the selective degradation of proteins associated with diseases. Additionally, this platform provides alternative avenues to explore druggable targets using targeting chimeras, rather than traditional inhibitory approaches (Winter et al., 2015). More recently, the use of artificial intelligence has greatly enhanced the rate at which potential degrader compounds can be identified and optimized allowing for the rational design of next-generation PROTACs that are both more efficient and more specific (Wang et al., 2025). In addition to chemical manipulation of ubiquitin-dependent signaling, there are families of E3 ligases, specifically tripartite motif (TRIM) proteins, which have been identified as key modulators of various cellular signaling pathways including innate immune response networks (Meroni and Diez‐Roux, 2005; Kawai and Akira, 2011). As such, the ubiquitin dependent immune regulatory function has broader implications for immunity, autophagy, and cancer development. These functions raise the possibility that engineered T cell–based therapies could potential also leverage ubiquitin-dependent immune regulation (Hatakeyama, 2017).

While significant advancements have been made, major challenges persist. Mechanisms regulating E3 ligases and their ability to select substrates and output of the signals based on context remain largely unclear. Therapeutic strategies currently target a very small fraction of the known E3 ligases; therefore, the remaining large portion of the ligase repertoire remains unexplored for clinical translation. Consequently, our understanding of E3 ubiquitin ligases remains fragmented across biological, pathological, and translational domains. The objective of this review is to integrate existing knowledge of E3 ubiquitin ligase biology with emerging therapeutic paradigms to provide a unified framework that links ubiquitin mediated regulation with the next wave of precision medicine.

## 2 Ubiquitination and classification of E3 ligases

**FIGURE 1:**
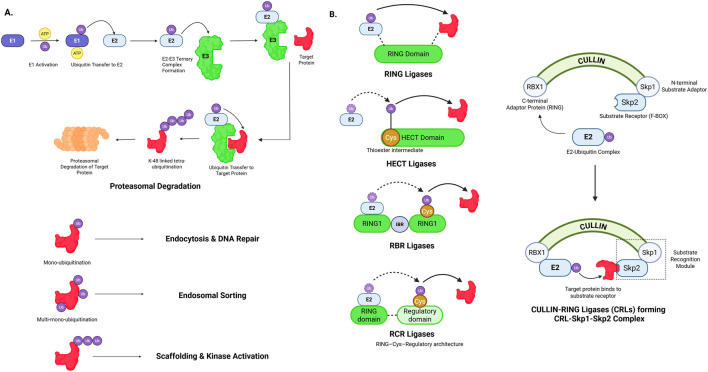
Mechanistic overview and classification of E3 ubiquitin ligases. **(A)** Canonical ubiquitination cascade in which E3 ligases catalyze substrate ubiquitination following E1 activation and E2 conjugation, leading to K48-linked polyubiquitination and proteasomal degradation, while mono- and multi-monoubiquitination mediate non-proteolytic signaling functions. **(B)** Structural and catalytic classes of E3 ligases, including RING, HECT, RBR, and RCR families, as well as multimeric Cullin–RING ligase (CRL) complexes with defined scaffold, adaptor, and substrate recognition modules. Created in BioRender. Krishnakumar, P. (2026) https://BioRender.com/zu2qjni.

The ubiquitination pathway is a three-step enzymatic process, as observed in Figure 1A, initiated by E1, the ubiquitin-activating enzyme, that forms a thioester linkage to Ubiquitin’s C-terminal glycine, thus activating ubiquitin. The activated ubiquitin is then transferred to E2, the ubiquitin conjugating enzyme, via transesterification. Finally, the ubiquitin is recruited from the ubiquitin-E2 complex by E3, the ubiquitin ligase. The recruited ubiquitin forms an isopeptide bond with the target protein, thus tagging the protein for clearance via UPS. Additionally, E4, the chain elongation ligases can extend already existing ubiquitin chains on the target protein. This pathway can tag proteins through monoubiquitination, multi-monoubiquitination or polyubiquitination. Mono-ubiquitination is the addition of a single ubiquitin molecule; multi-monoubiquitination is the attachment of multiple single ubiquitin molecules onto the substrate. Polyubiquitination is the attachment of whole ubiquitin chains onto the substrate. Based on their ubiquitin transfer mechanisms, E3 ligases are broadly classified into four major families: RING, HECT, RBR, and RCR E3 ligases (Berndsen and Wolberger, 2014; Swatek and Komander, 2016), as depicted in Figure 1B. U-box E3 ligases represent a specialized subclass of RING-type ligases that lack a canonical zinc-coordinating RING domain.

Ubiquitin is a 76 amino acid structurally conserved protein that has seven lysine residues (K6, K11, K27, K29, K33, K48, K63). This generates seven distinct lysine ubiquitination linkages and an eight linkage at the N-terminal methionine (M1) (Swatek and Komander, 2016). While the most occurring K48 linked polyubiquitination targets proteins for proteasomal degradation; the K11, K63 and M1 linked ubiquitinations (usually monoubiquitinations) regulate non-degradative processes like immune response, signal transduction and DNA repair. Hence distinct ubiquitination chain topologies determine the fate of target proteins. The versatile role of ubiquitin ligases makes them powerful therapeutic targets (Wang et al., 2013). For example, thalidomide functions as a molecular glue, reprograms substrate specificity of the CRL4-CRBN E3 ubiquitin ligase to target zinc-finger transcription factors (e.g., IKZF1, IKZF3, SALL4) for proteasomal degradation, thereby promoting therapeutic effects in multiple myeloma cells (Fraga et al., 2025). Another therapeutic strategy is proteolysis-targeting chimera (PROTAC), a bimolecular complex comprising a ligand-binding moiety linked to an enzyme-binding moiety via a linker. The enzyme binding molecule recruits the E3 ubiquitin ligase and helps target the previously undegradable proteins. The PROTAC dBET1 enhances apoptosis in leukemia, thus demonstrating anti-oncogenic properties (Winter et al., 2015). Bavdegalutamide (ARV-110) for prostate cancer, vepdegestrant (ARV-471) to target breast cancer, and KY-474 targeting autoimmune diseases are PROTACs under clinical trials. The PROTAC D16-M1P2 created using generative AI promotes anti-proliferation by degrading PKMYT1 that is lethal to *CCNE1*-amplified and *FBXW7*-mutated cancers (Wang et al., 2025). Recent advances in artificial intelligence have begun to support drug discovery by facilitating target identification and candidate optimization. In this review, we discuss emerging strategies that leverage the versatile roles of E3 ligases in therapeutic development, with an emphasis on minimizing cytotoxicity and oncogenic risk.

E3 ligases do not operate in isolation, but form regulatory hubs integrating multiple signaling inputs. In this review, we explore six interconnected functional domains: (Dikic, 2017): proteostasis maintenance through ERAD and chaperone-mediated quality control; (Finley, 2009); DNA damage responses requiring rapid, reversible ubiquitin signaling; (Wang et al., 2013); developmental fate decisions mediated by substrate switching; (Berndsen and Wolberger, 2014); signal transduction pathway control in stem cells and differentiation; (Swatek and Komander, 2016); cell cycle checkpoints enforced through oscillating cyclin degradation; and (Fraga et al., 2025) immune cell regulation balancing activation and tolerance. Understanding these functional relationships reveals why E3 ligase dysfunction manifests in oncogenic and neurodegenerative phenotypes. Table 1 discusses the major biological functions of the canonical E3 ligase families.

**TABLE 1:** Classification of major E3 ubiquitin ligase families based on catalytic mechanism, representative subfamilies, and core biological functions.

E3 ligase family	Catalytic mechanism	Major subfamilies	Major biological functions of the representative ligases	References
HECT E3 ligase	Ubiquitin is first transferred from E2 ligase to the E3 ligase’s HECT domain active-site cysteine, forming a thioester intermediate, and then transferred to the substrate	NEDD4 Subfamily: NEDD4, NEDD4L, ITCH (AIP4), WWP1 (AIP5), WWP2 (AIP2), NEDL1 (HECW1), NEDL2 (HECW2), SMURF1, SMURF2	SMURF1/2: degrade Mekk2, downregulate BMP and TGF-β signalling to inhibit osteoblast activity ITCH: Non-proteasomal degradation of TIEG1 maintains Treg function. Notch1 degradation inhibits Wnt signalling which then inhibits hematopoietic stem cell (HSC) proliferation WWP1: JunB degradation prevents mesenchymal stem cell (MSC) differentiation to osteoblast WWP2: OCT4 degradation maintains embryonic stem cell (ESC) pluripotency and fate NEDD4: Targets and degrades N-Myc and c-Myc, inhibit cell proliferation	Qian et al. (2020)
		HERC Subfamily: HERC 1 - 6	HERC1: Regulates cell proliferation by controlling C-RAF stability via K48-linked polyubiquitination. Regulates p38 MAPK pathway through MKK3. Regulates TSC2/tuberin HERC2: Facilitate RNF8-Ubc13 complex assembly that stabilizes RNF168 for damaged chromatin ubiquitination. Targets uncoupled BRCA1 for degradation preventing S phase checkpoint dysfunction.Facilitates MCM2 phosphorylation allowing DNA replication fork progression HERC3:K27 and K48 polyubiquitination of EIF5A2 preventing metastases of cancer, thus functioning as tumor suppressor HERC4: Promotes breast cancer by tumor suppressor LATS1 inhibition through miRNA interaction. Modest antiviral effects against HIV-1 HERC5: sustains innate antiviral immunity by maintaining ISG15 conjugation. Sustains IRF3 activation and boosts antiviral responses. Directly ISGylates viral proteins to disrupt replication (HCV NS5A at K379, SARS-CoV-2 N protein, HIV-1, influenza, Sendai virus, dengue). Regulates ISGylation-mediated reduction of host immunoproteins to prevent chronic inflammation HERC6: Promotes antiviral activity against vesicular stomatitis virus (VSV) and Newcastle disease virus (NDV); partially inhibits HIV-1. Regulates sperm sac morphology through ISGylation-independent mechanism	García-Cano et al. (2019)
		Other subfamilies	Ube3a/E6AP: Maintains synaptic plasticity and causes neurotoxic aggregate degradation, thus ensuring normal brain development HUWE1: mutations and dysregulation cause neurodevelopmental disorders via neuronal impairment, neuroinflammation and tumorigenesis	Ohno et al. (2025), Friez et al. (2016)
RING E3 ligase	Direct ubiquitin transfer from E2∼ubiquitin to the substrate without formation of an E3–ubiquitin intermediate. Catalysis is achieved by RING-mediated stabilization of a closed E2∼Ub conformation that optimizes ubiquitin transfer efficiency	Monomeric: Cbl Homodimeric: cIAP2 Heterodimeric: MDM2–MDMX; TRAF6–TRAF5 Multi-subunit complexes: BRCA1–BARD1 Cullin–RING ligase (CRL) complexes	Cbl (monomeric RING E3): Mediates ubiquitination of activated receptor tyrosine kinases to terminate growth-factor signaling and regulate signal amplitude and duration cIAP2 (homodimeric RING E3): Regulates cell survival pathways by controlling ubiquitin-dependent signaling in apoptosis and inflammatory responses, including non-degradative ubiquitin chain assembly MDM2–MDMX (heterodimeric RING E3): Controls cellular stress responses by regulating the stability and activity of the p53 tumor suppressor through ubiquitin-mediated turnover TRAF6–TRAF5 (heterodimeric/oligomeric RING E3s): Generate non-proteolytic ubiquitin chains that act as signaling scaffolds in innate and adaptive immune receptor pathways, activating NF-κB and MAPK signaling cascades BRCA1–BARD1 (multi-subunit RING E3 complex): Functions in chromatin-associated ubiquitination during the DNA damage response, contributing to genome surveillance and maintenance of genomic stability Cullin–RING ligase (CRL) complexes: Coordinate large-scale, substrate-specific ubiquitination programs that regulate cell-cycle progression, transcriptional control, and signal transduction across diverse cellular contexts	Metzger et al. (2012)
RBR E3 ligase	Hybrid RING–HECT–type mechanism in which ubiquitin is first transferred from the E2 enzyme to a catalytic cysteine within the RING2 domain, forming a transient E3–ubiquitin thioester intermediate before conjugation to the substrate	PARKIN HOIP (catalytic component of the LUBAC complex) HHARI (ARIH1)	RBR E3 ligases (general): Enable spatially and context-restricted ubiquitination by combining RING-type substrate recruitment with HECT-like catalytic chemistry, supporting precise regulation of signaling, stress responses, and protein quality control PARKIN: Regulates mitochondrial quality control by ubiquitinating outer mitochondrial membrane proteins, thereby promoting mitophagy and protecting cells from mitochondrial stress HOIP (LUBAC complex): Catalyzes linear (M1-linked) ubiquitin chain assembly to regulate inflammatory and immune signaling pathways, particularly NF-κB activation, without inducing proteasomal degradation HHARI/ARIH1: Functions as a catalytic E3 ligase that cooperates with Cullin–RING ligase complexes to elongate ubiquitin chains on CRL substrates, integrating scaffold-dependent substrate recognition with catalytic ubiquitin transfer	Spratt et al. (2014), Dove and Klevit (2017), Wenzel and Klevit (2012)
RCR E3 ligase	RING–Cys–Relay–type mechanism in which ubiquitin is transferred from the E2 enzyme to an active-site cysteine on the E3 ligase via an ester or thioester intermediate before final conjugation to the substrate. This mechanism is distinct from classical RING and RBR E3 ligases and enables non-canonical ubiquitin transfer chemistry	Non-canonical RCR-type E3 ligases identified through activity-based profiling, including esterification-competent E3 ligases	RCR E3 ligases (general): Mediate non-canonical ubiquitination reactions, including ester-linked ubiquitination, thereby expanding the chemical diversity of ubiquitin signaling beyond lysine-based isopeptide bonds and revealing additional layers of regulation in ubiquitin-mediated cellular control	Ikeda et al. (2011), Pao et al. (2018)
U-box E3 Ligase	Scaffold-type ubiquitin ligases that facilitate direct ubiquitin transfer from E2 enzymes to substrates without forming a covalent E3–ubiquitin intermediate. U-box domains adopt a RING-like fold but lack zinc coordination, relying on their structural configuration to position E2∼ubiquitin and substrates for efficient conjugation	CHIP (STUB1) PRP19	U-box E3 ligases (general): Dictate substrate specificity by selective pairing with E2 enzymes, enabling rapid and reversible ubiquitination in pathways such as DNA repair, proteostasis maintenance, and stress-response signaling CHIP (STUB1): Functions as a chaperone-associated E3 ligase that promotes ubiquitination of misfolded or damaged proteins, linking protein quality control to proteasomal and autophagic degradation pathways PRP19: Regulates DNA damage response and genome stability through ubiquitin-dependent control of repair-associated protein complexes and chromatin-associated processes	Shi et al. (2014), Idrissou and Maréchal (2022)

### 2.1 Emerging and non-canonical E3 ligase families

In addition to the canonical E3 ligase classes, new families have recently been described that expand classical E3 ligase classification frameworks. The new families differ from one another based on their regulatory mechanisms, localization, and the topology of ubiquitin chains, in addition to catalytic architecture, suggesting that the complexity of ubiquitin signaling is greater than previously thought.

One of the most widely studied and expanding subclasses of the RING-type E3 ligases is the TRIM family. TRIM proteins possess a conserved N-terminal RING domain, one or two B-box domains, and a coiled-coil region, followed by C-terminal regions of varying structure that determine their ability to bind to different substrates (Meroni and Diez‐Roux, 2005). Historically, TRIM proteins were considered typical RING-type ligases. However, many studies now indicate that TRIM proteins serve as signaling platforms for integrating ubiquitination with protein oligomerization and subcellular localization. As such, many TRIM proteins specifically catalyze the formation of non-proteolytic K63-linked ubiquitin chains and play key roles in various biological processes, including innate immunity, antiviral defense, chromatin remodeling, and cell cycle regulation (Kawai and Akira, 2011; Hatakeyama, 2017). The specialized functions of TRIM proteins establish this subclass as a separate family of RING-type E3 ligases.

Another new group includes E3 ligases that contain a PHD finger domain. These E3 ligases use PHD fingers, which are zinc finger domains with a “plant homeobox” structure, to facilitate ubiquitin transfer. Unlike RING domains, PHD fingers were originally identified as protein readers that recognize specific histone modifications. However, recent work has identified inherent E3 ligase activity in multiple PHD-containing proteins, including MEKK1 (MAP3K1) (Lu et al., 2002). PHD fingers can therefore read histones modified by post-translational modification and ubiquitinate the substrates simultaneously. This ability to both read chromatin modifications and ubiquitinate substrates directly relates the state of chromatin to the cell’s proteostasis and signaling output. The dual reader/writer capabilities of PHD finger containing E3 ligases distinguish them mechanically from RING containing E3 ligases and illustrate the emergence of another connection between epigenetics and ubiquitin mediated signaling.

The Ariadne (ARIH) family consists of several hybrid E3 ligases within the RBR superfamily that have been studied relatively recently. Although ARIH proteins are known to possess the RING1-IBR-RING2 architecture characteristic of all members of the RBR superfamily, ARIH proteins lack the ability to act as independent E3 ligases. Instead of working independently as RING or HECT type ligases, biochemical and structural studies have shown that ARIH ligases work cooperatively with Neddylated Cullin-RING Ligases (CRLs), by accepting ubiquitin from an E2 enzyme through a catalytic cysteine and elongating ubiquitin chains attached to CRL substrates (Duda et al., 2008; Scott et al., 2016). The cooperative mechanism described here represents an integration of the scaffold dependent recognition of the substrate with the catalytic addition of ubiquitin to the substrate, and therefore represents a non-canonical form of action for E3 ligases, bridging the actions of RING and HECT like E3 ligases.

In addition to the emerging E3 ligase families described above, several mechanistically well-defined E3 ligases have been shown to catalyze non-lysine ubiquitination reactions. A prominent example is MYCBP2 (also known as Phr1 or Highwire), a large E3 ubiquitin ligase that mediates ubiquitination on serine and threonine residues via ester-linked ubiquitin transfer. Biochemical and structural studies have demonstrated that MYCBP2 employs a cysteine-dependent relay mechanism to transfer ubiquitin prior to conjugation onto hydroxyl-containing amino acids on the substrate. This non-canonical ubiquitination activity plays a critical role in neuronal development and axon degeneration, establishing serine- and threonine-directed ubiquitination as a physiologically relevant E3 ligase–driven process (Pao et al., 2018; Kelsall, 2022).

RNF213 represents another well-characterized example of non-canonical E3 ligase activity. RNF213 is a giant E3 ligase with a dynein-like AAA + ATPase core that catalyzes ubiquitination through a distinct ubiquitin-transfer mechanism that is independent of classical RING-mediated catalysis. Mechanistically, RNF213 utilizes a cysteine-dependent ubiquitin transfer reaction that is distinct from classical RING-mediated catalysis and resembles relay-based mechanisms described for non-canonical E3 ligases. Importantly, RNF213 has been shown to ubiquitinate non-protein substrates, most notably the lipid A moiety of bacterial lipopolysaccharide (LPS), thereby initiating antibacterial immune signaling through downstream recruitment of ubiquitin-binding factors and linear ubiquitin chain assembly. Although the precise chemical linkage between ubiquitin and LPS remains to be fully resolved, these findings firmly establish RNF213 as a mechanistically defined non-canonical E3 ligase that expands ubiquitination beyond lysine residues and proteinaceous substrates (Kelsall, 2022; Otten et al., 2021; Ahel et al., 2020).

In addition to the families mentioned above, there are also higher-order assemblies of E3 ligases that are specialized in generating unique topologies of ubiquitin chains. These include the Linear Ubiquitin Chain Assembly Complex (LUBAC) consisting of HOIP, HOIL-1L and SHARPIN; and as a whole, LUBAC is responsible for forming M1 linked ubiquitin chains involved in signal transduction pathways that control inflammation and immunity, but do not lead to proteolytic degradation. This obligate assembly and chain specificity support the growing idea that E3 ligase classification is moving from being defined solely by catalytic domain architecture to being defined by the output of the function it performs and the regulatory structure of the ligase (Ikeda et al., 2011).

The RING-CH E3 ligases that are associated with cellular membranes (also known as March) have recently been recognized as an important family of E3 ligases. The March E3 ligases are identified based on their RING-CH domains and by having several transmembrane helices which place them at cellular membranes where they can ubiquitinate transmembrane proteins such as MHC molecules, immune receptors, and viral proteins [16]. While cytosolic RING ligases ubiquitinate soluble proteins destined to be degraded via the proteasome pathway, March E3 ligases control endocytic pathways, receptor internalization and lysosome targeting, providing evidence that the location of the E3 ligase is a key factor that defines the function of different E3 ligases.

In addition to lysine-directed ubiquitination, emerging evidence indicates that RING-CH (March) E3 ligases can also catalyze ubiquitination on serine and threonine residues of membrane-associated substrates. Viral and cellular RING-CH ligases have been shown to exploit hydroxyl-containing amino acids as ubiquitin acceptor sites on transmembrane immune receptors, including major histocompatibility complex (MHC) molecules, facilitating their extraction from membranes and subsequent degradation through ER-associated degradation pathways. These findings suggest that non-lysine ubiquitination may be particularly advantageous for membrane-embedded substrates, where lysine accessibility is constrained, and further highlight how subcellular localization and substrate topology influence E3 ligase catalytic strategies (Kelsall, 2022).

In addition to the Ubiquitin E3 ligases, there are other systems of Ubiquitin-like (UbL) E3s that provide additional mechanisms for the regulation of Post-Translational Modification of proteins. The SUMO E3 Ligase Family includes many well-known members such as PIAS Proteins, RanBP2 and ZNF451. These members of this protein family function by attaching small ubiquitin-like modifiers (SUMO) to the target proteins, which results in the regulation of transcription, DNA Repair and Nuclear Organization (Gareau and Lima, 2010; Johnson, 2004). While SUMO E3 ligases can operate in a mechanismally independent manner of Ubiquitin E3 ligases, they often work together with Ubiquitin Pathways and the concept of SUMO dependent Ubiquitination has emerged as one of the key regulatory axes for Genome Stability and Stress Response.

Collectively, the emerging E3 ligase families demonstrate that the modern classification of E3 ligases exceeds the confines of solely catalytic domain structure. As such, it will be increasingly important to incorporate the regulatory context of each E3 ligase family, as well as their chain specificity, subcellular localization, and ability to form cooperatively assembled complexes to fully capture the vast diversity in ubiquitin mediated signal transduction pathways. The recognition of the non-canonical E3 ligase families not only expands our understanding of the biological mechanisms involved in E3 ligase function, but also provides a larger pool of ligases which could potentially be used for therapeutic intervention.

## 3 Functions of E3 ligases and dysregulation

Substrate-specific ubiquitination by E3 ligases influences and integrates multiple signalling pathways. For example, loss of VHL (Von Hippel-Lindau) (i) promotes anti-tumor immunity through HIF1α/2α accumulation, which causes mitochondrial DNA leakage, and triggers cGAS-STING–interferon signaling axis (ii) downregulates ILC2 maturation by driving PKM2-mediated epigenetic suppression of the IL-33 receptor ST2. (iii) causes hyper-metabolism of myeloid derived suppressor cells (MDSCs) in the tumor microenvironment (TME) with enriched inflammatory response. While VHL loss initiates inflammation (via cGAS-STING) and slows tumoral growth, it exacerbates immune response by promoting MDSC metabolism leading to nutrient depletion, and immunosuppression in TME (Timms et al., 2023). These illustrate how E3 ubiquitin ligases contribute to cellular proteostasis, DNA damage repair, development and differentiation, and cell-cycle–linked cell fate decisions across diverse biological contexts. Hence, the dysregulation of an E3 ligase creates systemic dysfunction. To prevent this, we need to address its versatile role and determine possible therapeutic interventions. In Table 2, we discuss the known substrates of major E3 ligases and their associations to diseases.

**TABLE 2:** Representative E3 ubiquitin ligases, their molecular classes, key substrates or interaction partners, principal biological functions, and associated disease relevance.

E3 ligase	Type	Known substrates/Partners	Biological role/Pathway	Disease associations	References
UBR5	HECT (N-end rule)	β-Catenin; CDC73 (transcriptional regulators)	DNA repair, metabolism, apoptosis (controls turnover of regulatory proteins)	Overexpressed in breast, ovarian, and prostate cancers	Chen et al. (2021), Liao et al. (2017)
MDM2	RING (c-CBL family)	p53 (tumor suppressor); p21^Cip1/Waf1^ (cell-cycle inhibitor)	Negative feedback on p53 in DNA damage response; regulates cell cycle	Amplified or overactive in many cancers (leads to p53 degradation)	Haupt et al. (1997), Brooks and Gu (2006)
Parkin	RBR	Multiple outer mitochondrial membrane proteins (e.g., VDAC, mitofusins); NEMO (NF-κB modulator)	Mitophagy and mitochondrial quality control; links mitochondrial health to NF-κB signaling	Mutations cause autosomal recessive Parkinson’s disease; also implicated in Alzheimer’s (via mitochondrial dysfunction)	Yun et al. (2019)
CHIP	U-box (CHIP family)	HSP70/90-bound substrates (e.g., misfolded tau, amyloid precursor protein, mutant huntingtin)	Chaperone-assisted ubiquitination for proteasomal/autophagic degradation (proteostasis)	Dysregulation contributes to Parkinson’s, Alzheimer’s, and Huntington’s disease through impaired clearance of aggregates	Shi et al. (2014)
NEDD4/NEDD4-2	HECT (NEDD4 family)	Oncoproteins (N-Myc, c-Myc); viral matrix proteins (e.g., Ebola VP40); α-synuclein	Regulation of signaling (e.g., IGF, TGF-β); viral budding via ESCRT; neuronal proteostasis	Cancers (neuroblastoma, pancreatic); promotes viral egress (e.g., Ebola); linked to Parkinson’s via α-synuclein degradation	Hatstat et al. (2021)
ITCH	HECT (NEDD4 family)	Notch1 intracellular domain; JunB (AP-1 subunit); LATS1 (Hippo pathway)	Inhibits Notch signaling; regulates Hippo pathway; controls T‐helper cell differentiation	Immune dysregulation (e.g., ILC2 hyperplasia, Th2 skewing); cancer (via Notch/Hippo pathways)	Ho et al. (2011)
SMURF1	HECT (NEDD4 family)	BMP/Smad pathway components (Smad1/5, BMP receptors); RUNX2 (osteogenesis TF)	Inhibitor of BMP signaling; suppresses osteoblast differentiation (bone morphogenesis)	Metabolic bone diseases (osteoporosis) and impaired bone formation	Zhao et al. (2010)
WWP1	HECT (NEDD4 family)	LATS1 (Hippo pathway kinase)	Modulates Hippo signaling (negative regulator of LATS1); influences cell proliferation	Breast cancer (drives growth via LATS1 degradation)	Yeung et al. (2013)
Cbl-b	RING (Cbl family)	TCR signaling adapters (e.g., LAT, PLCγ1); PD-1 (indirectly)	Negative regulator of T cell activation; induces peripheral tolerance	Autoimmunity when deficient; targeting Cbl-b enhances CAR T-cell activity (tumor immunity)	Jeon et al. (2004)
c-Cbl	RING (Cbl family)	Receptor tyrosine kinases (EGFR, PDGFRβ, etc.)	Ubiquitinates and downregulates activated RTKs; regulates signal termination	Oncogenic when mutated (e.g., AML); dysregulated in cancers with RTK overactivation	Zuo et al. (2013)
FBXO38	F-box (SCF complex)	PD-1 (immune checkpoint receptor)	Ubiquitinates PD-1 for degradation, relieving T cell inhibition	Aids anti-tumor immunity; implicated in CAR T-cell exhaustion when absent	Meng et al. (2018)
RNF43	RING	Wnt receptors (Frizzled/LRP6)	Promotes endocytosis and degradation of Wnt co-receptors (negatively regulates Wnt/β-catenin signaling)	Tumor suppressor; inactivated by mutations in colorectal and other Wnt-driven cancers	Koo et al. (2012)
ZNRF3	RING	Wnt receptors (Frizzled/LRP6)	Similar to RNF43: downregulates Wnt signaling by targeting Wnt co-receptors	Tumor suppressor in Wnt pathway; mutations found in colorectal and pancreatic cancers	Hao et al. (2012)
RNF146	RING	Axin (upon PARylation)	Regulated by poly(ADP-ribose); mediates Axin degradation to modulate Wnt signaling	Impacts Wnt/β-catenin pathway; may influence cancer and developmental processes	Zhang et al. (2011)
RNF6	RING	Transducin-like enhancer protein 3 (TLE3; Wnt repressor)	Ubiquitinates TLE3 to enhance Wnt/β-catenin signaling; promotes cell proliferation	Overexpressed in colorectal cancer (oncogenic via Wnt activation)	Liu et al. (2018)
VHL	RING (CRL2 complex)	HIF-1α and HIF-2α (hypoxia-inducible factors)	Oxygen-dependent degradation of HIF; regulates hypoxic response; also controls immune cell development (T cells, ILCs)	von Hippel–Lindau syndrome; clear cell renal carcinoma; polycythemia when mutated	Galdeano et al. (2014), Diehl and Ciulli (2022)
RNF213	RING	Components of NF-κB signaling machinery; lipid-associated substrates	Regulates K63-linked ubiquitin signaling, vascular homeostasis, and inflammatory responses; functions as a mechanosensitive E3 ligase	Loss-of-function mutations cause Moyamoya disease; dysregulated inflammatory and angiogenic signaling	Mineharu and Miyamoto (2021)
MYCBP2 (also called Phr1)	RBR-like	Axonal and cytoskeletal regulators; neuronal stress-response proteins	Integrates axonal injury and stress signals to control ubiquitin-dependent neuronal remodeling and synaptic maintenance	Neurodevelopmental and neurodegenerative disorders (emerging evidence)	Babetto et al. (2013)

### 3.1 Cellular proteostasis

**FIGURE 2:**
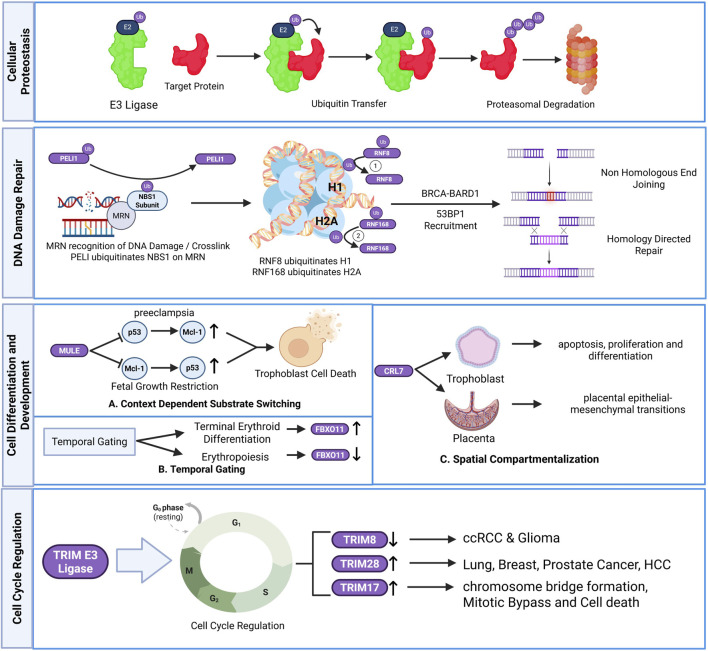
Context-dependent roles of E3 ubiquitin ligases in cellular physiology. E3 ligases regulate diverse biological processes through degradative and non-degradative ubiquitin signaling, including proteostasis, DNA damage repair, cell differentiation and development, spatial compartmentalization, and cell cycle control. Context-specific substrate selection, temporal regulation, and subcellular localization enable E3 ligases to coordinate distinct signaling outputs across physiological and pathological states. Created in BioRender. Krishnakumar, P. (2026) https://BioRender.com/nhezkyk.

The most fundamental role of E3 ligases is maintaining protein turnover. This role in ubiquitin-mediated proteasomal degradation is illustrated in Figure 2. Dysregulation of this activity can lead to oncogenesis, neurodevelopmental disorders and systemic cytotoxicity. Apart from those in Table 1, numerous E3 ligases are implicated in diseases. For example, in endoplasmic reticulum associated degradation (ERAD), ER localized E3 ligase Gp78 forms a complex with p97/VCP and Ube2g2 resulting in K48-linked polyubiquitination of mutant SOD1 and Ataxin-3 and their degradation (Van De Weijer et al., 2024; Van Den Boomen and Lehner, 2015). Thus, an exacerbated degradation response through Gp78 overexpression is therapeutic in neurodegeneration and cancer metastasis.

In another case of neurodegeneration, mitochondrial membrane potential (MMP) loss activates PARKIN which then induces mitochondrial surface remodelling by degradation of outer membrane proteins (Mfn1, Mfn2, Tom70, VDAC1, and Fis1) by K-48 linked polyubiquitination. Additionally, K-63 linked polyubiquitination by PARKIN recruits autophagy adaptors like p62/SQSTM1, and induces autophagy. Mutations in PARKIN cause onset of Parkinson’s Disease (PD) (Yun et al., 2019). Hence, activation of these E3 ligases is a neuroprotective strategy and their inhibition enhances anti-tumor effects by shutting down stress pathways for cell survival.

### 3.2 DNA repair

Another crucial role of E3 ligases is preventing genotoxicity through DNA repair. The sequential ubiquitination events governing DNA damage sensing and repair pathway choice are illustrated in Figure 2. Genomic DNA can be damaged by both exogenous and endogenous factors, generating single or double strand breaks (DSB), cross links of DNA strands and crosslinks with proteins. First, the DNA damage is detected by the MRN complex (MRE11-RAD50-NBS1) which in turn recruits ATM kinases through NBS1 (Koo et al., 2023). The NBS1 in the MRN complex is ubiquitinated by E3 ligases Peli1 and Skp2, this ubiquitination acts as recruitment signals for ATM kinase (Ha et al., 2019). The ATM kinase then phosphorylates H2AX to form γH2AX, attracting MDC1 that recruits RNF8 and Ubc13 (Wu et al., 2012). RNF8 ubiquitylates Histone H1 which acts as a signal and recruits RNF168 which causes mono-ubiquitination of Histone H2A at Lys 13/15 (Lee et al., 2016; Mattiroli et al., 2012). Ubiquitinated H2A recruits 53BP1 and the BRCA1-BARD1 to repair damaged chromatin by NHEJ or HDR.

However, RNF168 overexpression can be toxic to chromatin and is implicated in esophageal squamous cell carcinoma (ESCC) and malignancies (Yu et al., 2019). To prevent H2A over-ubiquitination, RNF168 is targeted for degradation by TRIP12 and UBR5 (Gudjonsson et al., 2012), while deubiquitinases like USP44 and OTUB1 remove excess ubiquitin markers (Mosbech et al., 2013; Wu et al., 2024). Enhancing these pathways to downregulate RNF168 is a viable therapeutic avenue for ESCC and RNF168 related genotoxic malignancies.

### 3.3 Development and differentiation of human cells

E3 ligases influence cell development decisions through three mechanistic strategies. These mechanisms are summarized in Figure 2. The first strategy - context dependent substrate switching exhibited by MULE. The E3 Ligase MULE regulates proteasomal degradation of Mcl-1 and p53. This activity determines trophoblast cell fate in placental disorders. In preeclampsia (PE), MULE mediated p53 degradation results in accumulation of apoptotic Mcl-1 variants. Conversely in fetal growth restriction (FGR), MULE stabilizes Mcl-1 resulting in p53 induced apoptosis. Dampening MULE’s either preferential effect can trigger worsening pathology of another (Sancho et al., 2022). Thus, developing therapeutics to target MULE is a challenge. However, ongoing investigations reveal that stabilization through deubiquitinase-targeting chimeras (DUBTACs) to remove ubiquitin chains on p53 or Mcl-1, can prevent exacerbated trophoblast cell death and is a novel therapeutic for E3 overexpression disorders (Chen et al., 2025).

The second strategy - spatial compartmentalization employed by CUL7. The specific localization of the E3 ligase to specific organelles controls the timing of ubiquitination and protein degradation. This means that E3 ligase is spatio-specific. For example, CRL7^Fbxw8^ regulates trophoblast by controlling apoptosis, proliferation and differentiation. Fbxw8 modulates p27 and cyclins through tumor suppressor BTG2 (Arai et al., 2003). Thus, it influences cell cycle progression. Meanwhile, CRL7^CUL7^ regulates placental epithelial-mesenchymal transitions (EMT) and development (Fu et al., 2010). Together, they influence decisions between normal and pathological placentation, especially in PE and FGR.

The third mechanistic strategy - temporal gating through E3 ligase regulation. During terminal erythroid differentiation, SCF^FBXO11^ is upregulated. However, during erythropoiesis, progenitor cells selectively degrade non-essential proteins to prioritize hemoglobin synthesis. The loss of FBXO11 elevates chromatin binding repressor BAHD1 levels. This in turn leads to reduced acetylation marks at erythroid gene promoters and interferes with GATA1 binding, which downregulates erythroid genes. Additionally, BAHD1 interacts with EZH2 and binds to H3K27me3 marks. This implies FBXO11 regulates polycomb group proteins through BAHD1. CRISPR screens show FBXO11 directly targets BAHD1 and inactivation of polycomb complexes partially rescues the differentiation defects, thus revealing a novel FBXO11-BAHD1-polycomb regulatory axis. Inhibiting this pathway can enhance erythropoiesis and reactivate fetal hemoglobin expression offering therapeutic promise for β-hemoglobinopathies (Xu et al., 2021).

In osteogenesis, E3 ligases influence bone formation by regulating mesenchymal stem cells (MSC) to osteoblast differentiation. SMURF1, WWP1, ITCH, Cbl-b and NEDD4 target Smad 1/5, BMP receptors, osteogenic transcription factors JUNB and Osterix. While SMURF1 inhibits osteoblast differentiation, NEDD4 and Arkadia (RNF111) promote osteoblast activity (Zhao et al., 2010). Similarly, SCFβ-TrCP and SMURF1 suppress osteogenesis through the Wnt/β-catenin pathway, while RNF146 enhances it. Additionally, c-Cbl and SPOP/SCFβ-TrCP regulate FGF and Hedgehog pathways (Umberger and Ogden, 2021). Moreover, ITCH degrades Notch intracellular domain (NICD), thus terminating notch signalling which enhances osteogenesis (Qiu et al., 2000; Chastagner et al., 2008).

The time-dependent exposure of parathyroid hormone exhibits dual effects. While continuous exposure increases SMURF1 levels that inhibits bone formation through RUNX2 degradation, intermittent exposure induces SCFβ-TrCP expression and stimulates osteogenesis (Liu et al., 2022). These insights reveal that time-dependent exposure of hormones can stimulate specific E3 ligases. This could provide a novel therapeutic strategy to treat metabolic bone diseases by promoting osteogenesis and bone regeneration, forming the basis of next-generation anabolic therapies for skeletal disorders.

### 3.4 Signal transduction pathways

The tumor heterogeneity of cancer stem cells (CSCs) is regulated by E3 ligase through signal transduction pathway modulation (Ciechanover and Kwon, 2015). For example, RNF43 and ZNRF3 degrade Wnt pathway co-receptors Frizzled and LRP6 which impairs signalling, thus regulating stemness (Koo et al., 2012). Additionally, WWP1 and ITCH degrade LATS1 which disrupts Hippo pathway, affecting CSC differentiation and their self-renewal characteristic (Yeung et al., 2013).

In another study, Wnt destruction complex destabilization through SIAH 1/2 mediated Axin degradation led to β-catenin accumulation, thus driving oncogenesis (Matsuzawa and Reed, 2001). Furthermore, RNF6 targets TLE3 which promotes β-catenin activity and enhances proliferation (Liu et al., 2018). Conversely, TRIM16 regulates Gli-1 in Hedgehog pathway that suppresses CSC traits (Tan et al., 2017). In addition, β-TrCP promotes β-catenin degradation under basal conditions keeping Wnt signalling in check. It can also regulate receptor availability through ZNRF3 turnover (Hao et al., 2012).

In DDR pathways, MDM2 downregulates p53. During DNA damage, p53 is stabilized and escapes MDM2 mediated degradation. However, rise in p53 levels activates MDM2 transcription creating a feedback loop. Dysregulation of this loop through MDM2 overexpression compromises DNA damage response and drives oncogenesis (Haupt et al., 1997). These findings highlight the significance of ubiquitination in cancer progression and CSC maintenance, suggesting potential anti-cancer therapeutic targets among E3 ligases.

### 3.5 Cell cycle regulation

In addition to other cellular processes, controlling the cell cycle (G1, S, G2, M) is fundamental to maintaining a healthy cell and dysregulating it contributes to many different diseases. Representative roles of TRIM E3 ligases in cell cycle regulation and cancer-associated dysregulation are illustrated in Figure 2. The TRIM family of proteins regulates the cell cycle through their ability to ubiquinate target proteins for degradation via the proteasome pathway. Specifically, the regulation of the G1/S checkpoint involves the ubiquination of proteins that are necessary for transition from G1 to S phase and the TRIM proteins involved in this process include TRIM52 (ubiquination of proteins involved in G1 to S transition), TRIM8 and TRIM28 (regulation of the p53-p21 axis). More specifically, TRIM8 stabilizes p53 leading to an increase in G1 arrest (Benke et al., 2018).

Given the involvement of TRIM proteins in the regulation of the cell cycle, it is not surprising that dysregulation of the expression of TRIM proteins is common in many types of cancers. For example, TRIM8 mRNA levels have been shown to be reduced in both ccRCC and glioma (Micale et al., 2015), whereas TRIM28 mRNA levels have been shown to be elevated in lung and breast cancer (Wei et al., 2016), as well as prostate cancer and HCC (Maghsoudloo et al., 2024).

In addition to regulating the cell cycle during interphase, TRIM proteins also play important roles in regulating mitosis and proper chromosome segregation. Both TRIM28 and TRIM32 are localized at centrosomes and spindle poles and appear to be important for maintaining proper mitotic fidelity (Izumi and Kaneko, 2014). TRIM36 and TRIM69 appear to play important roles in regulating kinetochore associated proteins to ensure proper chromosome alignment (Miyajima et al., 2009; Wang Y. et al., 2023). TRIM36 appears to regulate the CENP-H associated components to reduce the possibility of chromosomal instability, whereas TRIM17 appears to promote the degradation of the kinetochore protein ZWINT, which can lead to the formation of chromosome bridges, mitotic bypass and eventual cell death if it is not properly regulated (Endo et al., 2012). Together, these studies demonstrate that TRIM proteins play critical roles in regulating the cell cycle, maintaining proper mitotic integrity and ultimately reducing the potential for cancer development.

### 3.6 Immune regulation

In addition to regulating immune function as described in Section 1, ubiquitin ligases also help regulate immune response by either promoting or inhibiting the degradation of other important signaling proteins within the entire population of immune cells. The VHL E3 ligase regulates the immune system under low oxygen conditions, by way of regulating hypoxia-inducible factor (HIF) signaling, which influences both the development and function of immune cells (Doedens et al., 2013). Additionally, the HECT-type E3 ligase ITCH acts as an important regulator of immune homeostasis, to prevent excessive immune activation and promote a balanced response of T cells (Kathania et al., 2016). As regulators of immune homeostasis, E3 ligases integrate signals from the environment and cellular signals to maintain the balance of the immune system and influence inflammatory, autoimmune and neoplastic disease states. While E3 ligases do play a role in host-pathogen interactions, this review focuses primarily on their roles in immune regulation over pathogen-specific mechanisms.

## 4 Regulatory systems employed by E3 ligases in physiology and disease

Regulation of E3 ligase activity is mediated through a multi-layered system of regulation to ensure both precise catalysis and specific context. Instead of acting as continuously active enzymes, E3 ligases are used as coincidence detectors for signals, requiring multiple regulatory inputs prior to initiating ubiquitination. The system of regulation is based upon 4 separate yet interdependent systems: (Dikic, 2017): Covalent PTMs, (Finley, 2009), Signaling or small molecule–mediated recruitment of substrates, (Wang et al., 2013), Autoinhibition and Conformational Gating Mechanisms, and (Berndsen and Wolberger, 2014) Spatial and Temporal Compartmentalization of Ubiquitin Ligase Activity (De Bie and Ciechanover, 2011).

Each family of E3 ligases relies differently on each level of regulation which contributes to mechanistic diversity among E3 ligases. In contrast to rapid responses to PTMs and adaptor interactions by scaffold-type RING ligases, Catalytic HECT and RBR ligases must be unlocked through a series of structural transitions and receive multi-signal activation in order to initiate ubiquitination. An example of this is MDM2’s ability to mediate p53 degradation in response to a failure of three independent checkpoints including localization to the nucleus, productive p53 binding and lack of DNA damage signaling (Wenzel and Klevit, 2012). Therefore, disruption of an individual layer of regulation will produce distinct pathological conditions.

### 4.1 Post-translational modifications (PTMs)

PTMs are an important level of regulation that governs the activation of E3 ligases and their substrate specificity. Phosphorylation has been found to be the most common type of modification in RING-type E3 ligases. It appears to improve the positioning of E2 with ubiquitin and improve substrate association. For example, phosphorylated c-Cbl improves the ubiquitination of active receptor tyrosine kinase, and couples growth factor signaling with receptor downregulation (Zuo et al., 2013).

Neddylation is critical to the function of Cullin-RING ligases (CRLs). The conjugation of NEDD8 to cullin scaffold proteins creates a structural rearrangement that eliminates auto-inhibition and increases access of E2 to the ubiquitin, thereby increasing the rate of ubiquitin transfer (Enchev et al., 2015).

Additional levels of regulation exist beyond canonical ubiquitin based PTMs as well as additional types of noncanonical modifications. One such modification is the activation of RNF146 through the binding of poly(ADP-ribose) that links PARylation to ubiquitin mediated degradation of damaged DNA (Zhang et al., 2011). Auto-ubiquitination also serves as a regulatory mechanism for several E3 ligases, including cIAP1, where controlled self-ubiquitination modulates ligase stability and apoptotic signaling outputs.

### 4.2 Regulation by signaling molecules and small molecule effectors

Regulation of E3 ligase catalysis is generally achieved through extrinsic regulation of E3 ligase catalytic chemistry through extracellular and pharmacologic means or the modification of substrate recruitment; however, in plants, hormonal signals such as auxins and jasmonates can be considered molecular glue that promotes substrate binding by ligases such as TIR1 (Dharmasiri et al., 2005).

Therapeutic modulation of E3 ligase pathways have numerous implications for clinical applications in mammals. The drug bortezomib indirectly disrupts protein turnover dependent on E3 ligases causing an increase in levels of pro-apoptotic proteins which leads to selective apoptosis of cancer cells (Poulaki et al., 2007). This demonstrates the efficacy of selectively disrupting E3 ligase–substrate interactions to modulate ubiquitin signaling without directly inhibiting catalytic activity (Verma et al., 2010).

### 4.3 Autoinhibition, conformational regulation, and turnover control

E3 ligases have been shown to utilize intrinsic autoinhibitory mechanisms to maintain an inactive state when inappropriate, activating only when specific stimuli are present. For example, cIAP1’s activity can be inhibited through interaction of its BIR and RING domains, which upon apoptosis-induced signals are separated (Cheung et al., 2008).

The HECT ligase family members (e.g., NEDD4) require bilobed structures with flexible hinge areas to enable alignment of their catalytic residues; this can occur through phosphorylation or substrate engagement (Kamadurai et al., 2009).

Regulation of the RBR family of ligases is perhaps the most tightly controlled. For example, Parkin requires both PINK1-mediated phosphorylation, and phospho-ubiquitin binding to release it from autoinhibition and allow catalysis to occur only at damaged mitochondria (Wauer et al., 2015); demonstrating that E3 ligases may act as high fidelity coincidence detectors and thus demonstrate a new paradigm of regulation.

Similar to CRLs, regulation of E3 ligases also occurs through neddylation-dependent structural changes. In addition to activating E3 ligases, they also regulate the output of signaling pathways by determining the architecture of ubiquitin chains. For example, TRAF6 can assemble K63 linked chains of ubiquitin with the assistance of Ubc13 and recruit deubiquitinating enzymes (i.e., A20 and CYLD) to dynamically modulate NF-kappaB and MAPK signaling (Shi and Kehrl, 2010). Allosteric regulators such as SUMO and NEDD8 can also further stabilize the active conformations of E3 ligases (Liu and Nussinov, 2013).

### 4.4 Additional regulatory mechanisms

The use of adaptable F-box proteins and their common scaffold Skp1 allows for the definition of E3 ligase specificity, providing one mechanism of adaptor-mediated substrate selection in the CRL system (Skaar et al., 2013). The substrates are often recognized based upon the presence of specific degrons, which can include N-terminal degrons, C-terminal degrons, or internal structural degrons. These degrons are typically short sequence motifs or structural features whose accessibility and recognition are frequently regulated by post-translational modifications (PTMs); for example, phosphorylation of the β-catenin degron enhances its recognition by the SCF E3 ligase and promotes its subsequent degradation (Simonetta et al., 2019).

More recent research has demonstrated the importance of previously less understood and under-represented E3 ligase families. One such group consists of TRIM ligases, which contain RING, B-box, and coiled-coil domains and provide an integrated mechanism of regulating substrate oligomerization, localization and signaling. For example, the TRIM ligase TRIM25 regulates antiviral responses through the generation of K63-linked ubiquitin chains and illustrates how E3 ligases can be used to generate non-degradative forms of regulatory ubiquitin (Kawai and Akira, 2011).

Another regulatory paradigm for E3 ligases has received little attention and includes the Ariadne (ARIH) family of E3 ligases (e.g., ARIH1 and ARIH2) which are considered members of the RBR E3 ligase subfamily. However, structural and biochemical studies have demonstrated that ARIH proteins act as accessory E3 ligases when incorporated into Cullin–RING Ligase (CRL) complexes, whereas they do not possess catalytic activity independent of the assembled CRL scaffold. The CRL scaffold must be neddylated prior to the recruitment of ARIH for it to accept ubiquitin from E2 enzymes and lengthen substrate-linked ubiquitin chains. As such, this illustrates a hybrid regulatory mechanism that incorporates both scaffold-dependent control of CRL complexes and catalytic intermediate-based mechanisms typical of RBR type E3 ligases. This hybrid regulatory architecture positions ARIH ligases as conditional amplifiers of CRL activity, underscoring how higher-order complex assembly governs E3 ligase output (Scott et al., 2016).

A similar role for spatial organization has been found for the LUBAC, which utilizes the RBR ligase HOIP to produce M1-linked ubiquitin chains that are involved in the regulation of inflammatory signaling rather than proteasomal degradation (Ikeda et al., 2011). Like other E3 ligases, the activity of LUBAC is tightly regulated by the formation of a high molecular weight complex, and its localization, and the resulting chain topology serves as an additional level of regulation.

Additional layers of regulation have also been found to limit the spatial and temporal activity of E3 ligases. Spatial restriction further limits E3 ligase activity, as exemplified by Parkin, which is selectively recruited to damaged mitochondria where it initiates stress-responsive ubiquitin signaling (Brooks and Gu, 2006). In this context, Parkin-mediated ubiquitination coordinates both mitochondrial quality control and downstream signaling response (Narendra et al., 2008). In addition to mitophagy, Parkin-mediated ubiquitination also engages mitochondrial stress–induced inflammatory signaling pathways. Under conditions of mitochondrial damage, Parkin facilitates the recruitment and activation of NEMO (IKKγ), leading to downstream NF-κB activation. This Parkin–NEMO axis operates in parallel with mitophagy and highlights how E3 ligase activation can bifurcate into degradative and non-degradative signaling outputs depending on cellular context (Müller-Rischart et al., 2013).

As an additional layer of regulation, temporal control is provided by the ability of E3 ligases to become activated at distinct times. For example, the anaphase-promoting complex/cyclosome (APC/C) is activated during mitosis to regulate cell cycle progression. Furthermore, the circadian rhythm provides an additional level of temporal control over the activity of E3 ligases involved in the regulation of ubiquitin signaling (Abdalla et al., 2022). Finally, various environmental stresses such as hypoxia and oxidative stress activate certain E3 ligases (e.g., VHL, NEDD4, and HACE1) leading to aberrant regulation of inflammatory, metabolic and fibrotic pathways (Metzger et al., 2012).

Taken together, the results from studies examining the regulation of E3 ligase activity clearly indicate that the regulation of this class of enzymes is both localized and family-specific, and dependent on the cellular environment in order to ensure that ubiquitin signaling networks operate in a manner that is faithful to the physiological needs of the organism.

### 4.5 Dysregulation of E3 ligases in cancer

E3 ligases regulate oncogenesis through both degradative and non-degradative ubiquitin signaling mechanisms that collectively influence multiple hallmarks of cancer. While canonical K48-linked ubiquitination promotes proteasomal degradation of oncogenic or tumor-suppressive substrates, non-degradative ubiquitin signals such as K63-linked or linear chains modulate signaling amplitude, protein localization, and inflammatory responses without inducing protein turnover. Dysregulation of these ubiquitin outputs enables sustained proliferative signaling, evasion of apoptosis, and tumor-promoting inflammation (Swatek and Komander, 2016; Kawai and Akira, 2011).

Aberrant growth factor signaling, loss of contact inhibition, and defective feedback loops promote uncontrolled proliferation. By ubiquitinating regulatory proteins, E3 ligases influence both upstream receptor activity and downstream effectors, thereby determining the intensity and duration of mitogenic pathway.

Ligand binding to growth receptors activates receptor tyrosine kinase signalling that drives cell growth. However aberrant signalling can lead to uncontrolled proliferation of cells. E3 ligases prevent overexpression by ubiquitinating RTKs for degradation. The c-Cbl family ligases ubiquitinate and degrade numerous RTKs such as EGFR/ErbB1, PDGFR, NGFR/TrkA, FGFR2, HGFR/Met and CSF-1R (Wee and Wang, 2018). Meanwhile the TRIM21 regulates PDGFRβ turnover (Sarri et al., 2023). The ligase FBXW7 recognizes phosphodegron motifs on the EGFR tail and causes proteasomal degradation of EGFR. However, in colorectal cancer, mutations in the phosphodegron motifs or in FBXW7 leads to disruption in recognition (Yang et al., 2021). In another study, recruiting RNF167 to EGFR reduced cell proliferation in A431 squamous carcinoma cell lines. However, E3 ligases can also be oncogenic. In mesenchymal phenotype of gastric cancers, NEDD4 prevents PTEN’s dephosphorylation of IRS1 which in turn keeps downstream signalling active. This causes IGFR1 overexpression. Targeting NEDD4 degradation is an alternate therapeutic strategy when IGFR1 inhibitors fail (Wang K. et al., 2023). The role of E3 ligases further extends to the MAPK and PI3K-AKT downstream signal transduction pathways of RTKs. Importantly, while ligases such as c-Cbl and FBXW7 suppress oncogenic signaling through degradative ubiquitination, ligases such as NEDD4 and TRAF6 promote tumorigenesis through non-degradative ubiquitin signaling that sustains downstream pathway activation.

Downstream of RTKs, the CRL3 E3 ligase - LZTR1 ubiquitinates Ras proteins which downregulates RAS/MAPK pathway. The BTB domain of LZTR1 binds to CUL3 and the Kelch domain binds to the Ras proteins. CUL3 then attaches K-48 linked ubiquitin chains to the Ras proteins. Loss of function mutations in LZTR1 result in RAS/MAPK-driven cancers. In another study, LZTR1 causes degradation of small RAS-GTPase RIT1. However, oncogenic mutations on RIT1 prevent LZTR1 mediated proteolysis and thus causing developmental disorders like Noonan Syndrome (Steklov et al., 2018). Similarly, TRAF6 ubiquitinates PI3K catalytic subunit PIK3CA that causes PI3K activation. However, its overexpression is an oncogenic mechanism (Wang et al., 2018). This highlights how non-proteolytic ubiquitin modifications can act in parallel with degradative pathways to amplify oncogenic signaling outputs.

CRL1 and APC/C complexes act throughout the cycle to remove inhibitors and ensure correct timing (Nakayama and Nakayama, 2006). CRL4AMBRA1 targets Cyclin D1 for degradation, restricting unscheduled G1/S transition, whereas SCFSkp2 eliminates CDK inhibitors like p27Kip1 to enable S-phase entry (Simoneschi et al., 2021). APC/CCDC20 and APC/CCDH1 regulate chromatid segregation and mitotic exit, maintaining post-mitotic stability (Hu et al., 2011). E3 ligases thereby preserve cell-cycle fidelity, yet their mutation or misregulation promotes oncogenic proliferation. FBXW7 is a haploinsufficient tumor suppressor that normally degrades Cyclin E, c-MYC, and NOTCH. Its loss in cholangiocarcinoma and T-cell acute lymphoblastic leukemia (T-ALL) destabilizes cell-cycle checkpoints. Conversely, in multiple myeloma FBXW7α can act in a pro-survival manner by degrading the NF-κB inhibitor p100. Cyclin D1 accumulation, frequent in breast, lung, and prostate cancers, often arises from defective degradation. AMBRA1 mutations stabilize D-type cyclins, as shown in murine models (Simoneschi et al., 2021). CRL1Skp2 acts oncogenically by degrading the CDK inhibitor p27, thereby hyperactivating CDK1/2; it is often amplified in prostate cancer and lymphomas (Kossatz et al., 2004). However, Skp2 is crucial for hepatocyte proliferation and maintaining hematopoietic stem cell populations which makes Skp2 mediated therapeutic development challenging (William et al., 2024). By contrast, CRL1βTrCP has context-dependent roles: it can act as a tumor suppressor in some settings, yet is overexpressed in colorectal, hepatoblastoma, and breast cancers and has rare activating mutations in gastric and prostate cancers (Frescas and Pagano, 2008).

To combat oncogenic developments, apoptosis serves as a natural barrier to malignancies. However, its evasion can drive oncogenesis. The E3 ligases regulate both intrinsic and extrinsic apoptotic pathways by maintaining a balance of pro- and anti-apoptotic factors. Ideally, an increased proliferation of cells due to oncogenic developments in growth signalling should be met with increased apoptotic demand. However, evidence suggests that proliferative growth of cells outpace apoptotic mechanisms leading to tumorigenesis and malignancies (Pistritto et al., 2016).

E3 ligases control mitochondrial apoptosis by regulating BCL2-family proteins that keep pro-apoptotic proteins in check, thereby maintaining mitochondrial membrane integrity. Cancer cells often subvert this control through RAS–ERK and PI3K–AKT pathways. CRL1βTrCP promotes survival by degrading pro-apoptotic BIM1 following phosphorylation on serine residues in phosphodegron motifs, while CRL1FBXO25 targets anti-apoptotic HAX-1 for proteasomal degradation. However, deficiency of HAX1 results in severe neutropenias and diminished contractile function of cardiac muscles due to SERCA2a degradation (Bidwell et al., 2018). FBXO25 deletion, common in lymphomas, overexpresses HAX-1 and confers resistance to apoptosis (Cai et al., 2024). The inhibitor of apoptosis protein (IAP) XIAP, a RING-type E3, directly ubiquitylates and inhibits caspases-3, 7, and 9, blocking apoptotic execution.

While extrinsic apoptosis is triggered by death receptors (e.g., TNF, TRAIL) that form the DISC complex to activate caspase-8, inhibitor of apoptosis proteins (IAPs), including cIAP1/2, suppress this pathway by K63-linked polyubiquitination RIP1 to promote cell survival. Overexpression of cIAP2 prevents ripoptosome formation, thus driving cells away from apoptosis and necroptosis, in pancreatic cancer, resulting in chemoresistance and poor prognosis (Pistritto et al., 2016).

One well-characterized example of apoptosis evasion involves p53 inhibition. The tumor suppressor p53 is chiefly regulated by the RING E3 MDM2, which maintains low basal p53 levels through continuous ubiquitination and degradation. Genotoxic stress disrupts this interaction, stabilizing p53 to activate genes mediating cell-cycle arrest, apoptosis, and DNA repair (Haupt et al., 1997). MDM2 amplification, a frequent oncogenic event, thus suppresses p53 and promotes tumorigenesis (Pistritto et al., 2016).

Apart from oncogenic mutations on growth receptors, the downstream signalling of RTKs, the cell cycle checkpoints and apoptotic pathways, DNA damage response (DDR) addressing E3 ligases that ensure genomic integrity are potential oncogenic drivers. The highly genotoxic double stranded breaks (DSB) are repaired using RING type E3 ligases. RNF8 recruited to the damage site synthesizes K63-linked ubiquitin chains on proximal H1, H2A and H2AX histones. This ubiquitin mark signals RNF168 recruitment, that monoubiquitinates K13 and K15 residues on H2A (Huen et al., 2007). The monoubiquitination mark signals the recruitment of BRCA1-BARD1 complex (Wang et al., 2007). The entire mechanism promotes homologous recombination. However, during cancer, oncogenic mutations often disrupt this process leading to accelerated tumorigenesis and inflammation (Chauhan et al., 2024).

Beyond DDR, E3 ligases influence DNA repair enzyme bioavailability. SCFCyclin F targets the ribonucleotide reductase component RRM2 for degradation by K-48 linked polyubiquitination, thus preventing dNTP overproduction after the S phase of the cell cycle. Meanwhile, APC/CCdh1 maintains low RRM2 in G1 and SCFCyclin F clears it in G2. Cyclin F also targets activator E2Fs, thus controlling G1 to S transition. However, under genotoxic stress, Cyclin F downregulation by ATR checkpoint activation increases RRM2, enabling dNTP production for DNA repair. Evidence suggests oncogenic mutations in Cyclin F cause cell cycle disruption. Studies show loss of function mutation in Cyclin F causes E2F accumulation resulting in aberrant transcription of proliferative genes like c-Myc in Burkitt lymphoma. In another case, Cyclin F dysregulation causes familial Hodgkin lymphoma (fHL) (Khoury et al., 2023).

While dysregulation of E3 ligases initiate malignancies, it causes tumorigenesis that is supported by angiogenesis. The central regulator of angiogenesis is HIF composed of two subunits - the oxygen sensitive HIFα and β. Under normoxia, hydroxylated HIFα is proteasomally degraded by the VHL complex. However, hypoxia inhibits prolyl hydroxylase domains (PHD) which aids recognition of HIF by VHL. This stabilizes HIFα levels and allows dimerization with HIFβ which induces VEGF, EPO, GLUT1 and angiopoietin transcription. This promotes neovascularization. A loss of function mutation in VHL is a hallmark of von Hippel Lindau syndrome and clear cell renal carcinomas. While inhibiting the VHL-HIF-VEGF axis using VEGF inhibitors bevacizumab and TKIs sunitinib (Motzer et al., 2007) and sorafenib (Escudier et al., 2007), cancerous cells gain resistance through compensatory pathways (Li and Ramli, 2025).

These versatile roles of E3 ligases show immense potential in anti-cancer therapeutics. Recent advancements in delivery systems have further enhanced the latent potential. For example, utilizing radiolytic *Escherichia coli* Nissle injections to deliver the bacteria with the therapeutic gene insert directly to tumor sites. The facultative anaerobic nature of the strain allows growth within hypoxic TME and allows continuous release of the therapeutic after radiation (Kamble et al., 2022). This novel delivery technique could further be enhanced by allowing the strain to synthesize BioPROTACs, a novel proteasomal degradation method that can be therapeutic in not only cancer, but numerous disorders caused due to E3 ligase dysregulation.

Collectively, these examples demonstrate that E3 ligase dysregulation converges on multiple hallmarks of cancer, including sustained proliferative signaling, evasion of apoptosis, genomic instability, tumor-promoting inflammation, and angiogenesis. By functioning at the intersection of growth factor signaling, cell-cycle control, DNA damage repair, metabolic adaptation, and immune modulation, E3 ligases act as central regulatory nodes in oncogenesis. This convergence positions E3 ligases as compelling therapeutic targets capable of simultaneously modulating multiple cancer-driving processes.

### 4.6 Dysregulation of E3 ligases in neurodegenerative disease

The accumulation of misfolded and aggregated proteins: ɑ-synuclein in Parkinson’s disease (PD), amyloid β (Aβ) in Alzheimer’s disease (AD), and Huntingtin in Huntington’s disease (HD) drive progressive neurodegeneration. This also functions as damage associated molecular patterns (DAMPs) that drive neuroinflammation. E3 ligase dysregulation results in a cycle of protein accumulation, neurodegeneration and neuroinflammation (Liu et al., 2023).

#### 4.6.1 E3 ligase dysregulation in PD

The accumulation of presynaptic protein ɑ-synuclein causes progressive neurodegeneration of the substantia nigra dopaminergic neurons. The U-box containing E3 Ligase CHIP’s TPR domain facilitates proteasomal and lysosomal degradation of pre-synaptic protein ɑ-synuclein through Hsp70/Hsp90 mediation. Contrastingly, NEDD4 ubiquitinates ɑ-synuclein directly. This results in endosomal trafficking of this and aggregation into Lewy bodies (Tofaris et al., 2011). Evidences show that NEDD4 inhibition using N-aryl benzimidazole reduces ɑ-synuclein aggregates that results in reduced toxicity in PD models (Hatstat et al., 2021). Moreover, a novel BioPROTAC for ɑ-synuclein using CHIP bound to NbSyn87, a nanobody exclusive to ɑ-synuclein shows immense anti-PD potential warranting further testing (O’Shea and Wright, 2025).

Additionally, mitochondrial dysfunction also drives PD pathogenesis. PINK1 recruits parkin that ubiquitinates outer mitochondrial membrane proteins. This initiates mitophagic clearance that prevents release of mitochondrial DAMPs. In addition, parkin also initiates inflammatory response by linearly ubiquitinating NEMO. This upregulates the neuroprotective NF-κB pathway (Matsuda et al., 2010). In activated microglial cells, parkin suppresses LPS induced NF-κB activation and tags NOD2 for degradation, driving down inflammatory response (Yun et al., 2019). However, mutations in PARK2 gene observed in early onset of PD result in loss in function of parkin which amplifies neurodegeneration and neuroinflammation. In another case, PD-linked protein LRKK2 signals C3HC4, March5, MULAN and parkin to localize to the mitochondria and ubiquitinate mitochondrial membrane components. However, CHIP ubiquitinates LRKK2 which makes CHIP an ideal therapeutic target (Burchell et al., 2013). This suggests parkin upregulation as a significant therapeutic strategy using small molecular activators (cinnamon, lumateperone), inhibiting parkin inhibitors (c-Abl, USP30/33 inhibitors) and upregulating other mitophagy promoters such as NIX in cases with PARK2 mutations.

#### 4.6.2 E3 ligase dysregulation in AD

Accumulation of Aβ plaques and hyperphosphorylated tau neurofibrillary tangles accompanied by TDP-43 accumulation causes AD. CHIP inhibits Aβ production by targeting amyloid precursor protein for degradation. Additionally, NRBP1 in the Cullin RING complex inhibits Aβ biosynthesis by targeting ITM2B and BR13 for degradation (Yasukawa et al., 2020). Another neuroprotective ligase PIAS1 sumoylates amyloid precursor protein intracellular domain which promotes neprilysin and transthyretin expression, consequently increasing Aβ degradation (Liu Y-C. et al., 2021). At the same time, sumoylation of TDP-43 maintains its solubility in cytosol, thus preventing aggregation (Pendlebury et al., 2025). Recent studies establish Praja1’s role in TDP-43 degradation and maintaining synaptic plasticity (Li et al., 2025). Furthermore, PELI1 plays a crucial role in microglial-mediated Aβ phagocytosis. Dysregulation of PELI1 compromises plaque clearance and exacerbates neuroinflammation, highlighting its relevance in disease progression (Xu et al., 2020). Hence, we can conclusively say that in a normal phenotype, E3 ligases play a neuroprotective role.

However, in AD, there is cascadal dysregulation of E3 ligases. CHIP, Praja1 and UBE3a are significantly downregulated causing Aβ and tau accumulations. TRAF6 plays a dichotomous role of promoting neuroinflammation by NF-κB pathway, but paradoxically ensuring neuronal survival and spinogenesis. Ube3a downregulation resulted in PPAR-α mediated ADAM10 upregulation that decreased Aβ levels. But it also inhibited the neuroprotective ESR2 gene transcription leading to motor and cognitive functional loss as observed in Angelman syndrome. Parkin deficiency results in mitochondrial dysregulation. However, while parkin is expressed normally, specific truncated tau can recruit Parkin, causing excessive mitophagy and neuronal loss (Potjewyd and Axtman, 2021). While bolstering E3 ligase activity is a therapeutic intervention for AD, the off-target synaptic and metabolic deficits need to be addressed.

#### 4.6.3 E3 ligase dysregulation in HD

HD is an autosomal dominant neurodegenerative disorder caused due to CAG repeat expansion in the huntingtin gene resulting in production of mutant huntingtin (mHTT). Its aggregation causes neuronal toxicity. In astrocytes, CHIP tags mHTT for proteasomal degradation. However, in neurons, this is inhibited by HspB1. This implies that mHTT degradation is cell type specific. Additionally, UBR5 promotes mHTT degradation (Koyuncu et al., 2018). In another study, PIAS1 reduction rescued the neurons from mHTT aggregation and increased their survival rate. Furthermore, PIAS variants have been implicated in polyglutamine alterations. Alternatively, SCF inhibition resulted in CUL1 mediated mHTT aggregation. Conversely, the SCF complex component FBXW7 degrades CHK2 and p53, thus reducing DNA repair response and exacerbating mitochondrial stress. It also results in heat shock factor 1 (HSF1) degradation that increases neuronal stress (Kang et al., 2025). This implies that E3 ligases play a role that is specific to cell types in both neuroprotection as well as degradation. Furthermore, UBE2K E2 Ligase is implicated in polyglutamine expansions. Thus, inhibiting UBE2K is a potential therapeutic avenue that warrants investigation.

Other studies from postmortem brains with HD show WWP1 upregulation in HD causes K63-linked ubiquitination of mHTT. Furthermore, TRAF6 upregulation causes K6, K27 and K29 linked ubiquitinations. These upregulations inhibit mHTT degradation and potentially promote inflammatory signalling. Additionally, PIAS1 is directly implicated in neuroinflammation in HD (Morozko et al., 2021). In another study, SOCS2 upregulation in striatal cells downregulates apoptosis and continuous mHTT accumulation (Liu X-Y. et al., 2021). These suggest downregulation of WWP1, TRAF6, PIAS1 and SOCS2 are therapeutic interventions to restore protein clearance, apoptosis mechanisms and reduce neuroinflammation in HD [103].

Across PD, AD, and HD, a common failure mode emerges: E3 ligases like CHIP become substrates of the very aggregates they are meant to clear. In HD models, mHTT aggregates sequester CHIP, preventing it from ubiquitinating other misfolded proteins, a dominant-negative effect. Similarly, α-synuclein oligomers in PD inhibit the 26S proteasome, creating a proteostatic bottleneck even when E3 activity remains intact. This explains why single-target approaches fail: restoring CHIP expression without addressing aggregate-mediated sequestration or proteasome inhibition provides no benefit. Therapeutic strategies must therefore address both E3 ligase activity and aggregate clearance, potentially via bifunctional molecules capable of activating E3 ligases while facilitating aggregate disassembly.

### 4.7 E3 ligase dysregulation in genetic disorders

Genetic mutations that result in gain of function and loss of function of E3 ligases can impact critical developmental processes such as neurodevelopment in Autism spectrum disorder, Angelman’s syndrome and many more. Additionally, it can result in severe immunodeficiency as observed in Fanconi Anemia and RIDDLE syndrome. Also, it can increase the risk of cancers and strokes by increasing mutation rates and genotoxic stress. Rare diseases such as those caused by E3 ligase mutations seldom have disease modifying therapies that are available as observed in Table 3, which highlights some genetic disorders caused due to E3 ligase dysregulation.

**TABLE 3:** Neurodevelopmental–neurological and genetic disorders associated with dysregulation of E3 ubiquitin ligases.

Disease	Dysregulated E3 ligase	Biological implication	Therapeutic intervention	References
Neurodevelopmental–neurological disorders				
Autism spectrum disorder (ASD)	Gain of Function in UBE3A	In neurons, hyperactive mutations, and duplication of maternal UBE3A gene 15q11-q13 chromosome region causes synaptic protein disbalance leading to abnormal dendritic spine development and altered synaptic plasticity. Hyperactive UBE3A is also implicated in astrocytes	Reducing UBE3A activity using protein kinase A (PKA) mediated inhibition and antisense oligonucleotide therapeutics to decrease UBE3A mRNA levels	Ohno et al. (2025)
	Loss of function in CUL3	Nonsense and missense mutations in CUL3 cause RhoA buildup. This dysregulates dendrite formation, enhances glutamatergic impulse transmission, and impairs synaptic transmission	RhoA or eIF4G1 inhibition partially rescues cellular functions	Ohno et al. (2025), Sekar et al. (2025)
	Loss of function in TRIM32	Deletions in ASTN2/TRIM32 leads to reduced GABAergic interneurons due to neural progenitor cell self-renewal impairment, suppressed mTOR and Notch signalling, altered synaptic plasticity, excitation-inhibition imbalance	Small molecule mTOR activator 3BDO rescues impaired interneuron generation. GABA receptor agonist Clonazepam rescues hyperexcitability.	Zhu et al. (2020), Thomas et al. (2023)
Angelman’s syndrome (AS)	Loss of function in UBE3A	Maternal loss of function causes Arc protein buildup leading to synaptic dysfunction. Accumulation of p53 and Ephexin5 disrupts neural development	Blocking UBE3A-ATS lncRNA to activate paternal copy of UBE3A gene using antisense oligonucleotide therapeutics and CRISPR.	Bossuyt et al. (2021)
Juberg- Marsidi Syndrome (JMS)	Loss of function in HUWE1	Missense mutation p.G4310R in HUWE1 gene leads to p53 and Mcl-1 accumulation that impairs neural differentiation. This causes impaired neurodevelopment characterized by severe X-linked intellectual disability	Reducing p53 levels can rescue neural differentiation allowing normal brain development	Friez et al. (2016)
Clark- baraitser syndrome (CBS)	Loss of function in TRIP12	Failure in normal ubiquitination of PARP1 and NRF2 leads to oxidative stress in neural tissues and muscles leading to dysmorphic facial features, motor and speech impairment and intellectual disability	Reducing PARP1 and NRF2 levels rescues cells from oxidative stress allowing normal p53 pathways to resume	Van Der Laan et al. (2022)
Gordon holmes syndrome (GHS)	Loss of function in RNF216	Accumulation of cytoskeletal protein Arc/Arg leads to synaptic dysfunction causing cognitive decline and cerebellar ataxia. Additionally affects the hypothalamus-pituitary-gonadal (HPG) axis causing inadequate sexual development	Gonadotropin stimulation and anticholinergics for symptom management. Combinatorial therapy with anti-seizure drugs, hormone replacement therapeutics and deep brain stimulation shows good effect	Cotton et al. (2022)
	Loss of function in CHIP	Causes motor and reproductive impairments causing ataxia and hypogonadotropic hypogonadism		Shi et al. (2014)
Moyamoya disease (MMD)	Loss of function in RNF213	Impairment in K63 linked polyubiquitin chain production causes enhanced NF-кB inflammatory signalling and increased apoptosis. It also causes abnormal angiogenesis resulting increased risk of strokes	No disease modifying therapy available	Mineharu and Miyamoto (2021)
Genetic disorders				
Fanconi anemia (FA)	Loss of function in FANCL	Majorly affects the bone marrow and HSC populations. Failure of DNA interstrand crosslink repair leads to increased pancytopenia. Additionally, impaired mitophagy causes increased oxidative stress and inflammatory signalling. It also contributes to tumor progression by increasing mutation rates, especially leukemia	No disease modifying therapy available. HSC transplantation increases survival rates of patients. Antibody based conditioning using JSP-191 is under clinical trials	Cancio et al. (2024)
RIDDLE syndrome	Loss of function in RNF168	Nonfunctional RNF168 prevents amplification of H2A/H2AX ubiquitination signals. Thus, 53BP1 foci formation is impaired which prevents DSB repair. This causes reduced immunoglobulin levels and immunodeficiency	No disease modifying therapy available. Combination of Symptom management therapy and Immunoglobulin replacement therapy is a symptom management therapeutic intervention	Kelliher et al. (2022)
Johanson- blizzard syndrome (JBS)	Loss of function in UBR1	Affects pancreatic acinar cells causing severe congenital exocrine pancreatic insufficiency and onset of destructive pancreatitis	No disease modifying therapy available	Zenker et al. (2005)
Opitz G/BBB syndrome (OS)	Loss of function in MID1	Decreased midline-1 (MID1) function results in PPA2c accumulation that leads to microtubule apparatus assembly impairment. This results in cardiac, craniofacial, Laryngotracheoesophageal and neurological defects	No disease modifying therapy available	Quaderi et al. (1997)

Footnote–Diseases are classified as neurodevelopmental–neurological when nervous system dysfunction represents the primary phenotype; remaining conditions are grouped as genetically defined multisystem disorders.

Most mutations in E3 ligase cause proteinopathies leading to increased oxidative stress and ultimately apoptosis or an inflammatory signalling. As observed in Table 3, neurodevelopmental–neurological conditions such as ASD, AS, JMS, CBS, and GHS arise from the deficiency or accumulation of neuronal proteins, leading to disrupted synaptic plasticity and impaired signaling. For example, the RBR E3 ligase ARIH2 interacts with E2 ligase UBE2L3 and functions like HECT ligases. It directly influences hedgehog signalling required for myelopoiesis during embryonic development. Specifically, it regulates degradation of smoothened protein in the endoplasmic reticulum which is essential for neural differentiation. ARIH2 has been identified to function as a co-E3 ligase and to regulate the Cullin-RING ligase (CRL) activity via monoubiquitination of DCNL1, which affects the ubiquitin dependent signaling pathways that are important for developmental processes. The dysregulation of these CRL remodeling mechanisms can also affect the neurodevelopmental signaling pathways. Hence, in neurodevelopmental disorders such as ASD, ARIH2 dysregulation might play a role in exacerbating neurodevelopment impairment (Kelsall et al., 2019).

Comparing the role of UBE3A in Angelman Syndrome and ASD from Table 3, the importance of balanced E3 Ligase activity is highlighted. Loss of function in the maternal UBE3A gene leads to Angelman Syndrome. Conversely, duplication or triplication of this gene causes autism in ∼1–3% of cases. This establishes UBE3A as a critical therapeutic target and an underlying principle - the normal levels of E3 ligase mimic ‘Goldilocks Zone’, implying that regulation of normal E3 ligase levels is crucial for normal functioning of the body. This parallels FBXW7s oncogenic role - haploinsufficiency drives T-ALL through NOTCH1 and c-Myc accumulations, while complete deficiency of FBXW7 is embryonically lethal. Quantitative modelling reveals the therapeutic window of E3 ligase modulators is significantly narrower than small molecule enzyme inhibitors. To maintain E3 ligase balance using short-acting modulators demands consistent and patient specific dosing highlighting the role of precision medicine in E3 therapeutics (Amar et al., 2021).

Taken together, genetic disorders caused by E3 ligase dysregulation underscore the requirement for precise quantitative control of ubiquitin signaling during development and tissue homeostasis. Both gain- and loss-of-function mutations disrupt tightly balanced proteostasis networks, leading to neurodevelopmental, immunological, and metabolic pathologies. These observations highlight that E3 ligases operate within narrow therapeutic windows, where partial modulation may be beneficial but complete inhibition or overactivation can be deleterious. Consequently, future therapeutic strategies must prioritize precision dosing, temporal control, and tissue specificity when targeting E3 ligases in inherited disease.

## 5 Clinical translation: E3 ligases as programmable effectors for therapeutics

Recent developments in therapeutics have established E3 ligases as being programmable molecular effectors in selective protein degradation. E3 ligase-mutated ubiquitination is now being used as both small-molecule inhibitors as well as genetically engineered cell therapies, dependent on the context. PROTACs and CAR T-cell engineering are different inherently in terms of delivery and implementation. However, they are mechanistically similar in that they depend on substrate redirection instead of enzymatic inhibition. Hence, E3 ligases form a crucial cog in the machinery of next-generation therapeutic strategies.

### 5.1 E3 ligases as recruitable effectors in PROTAC-Based drug development

**FIGURE 3:**
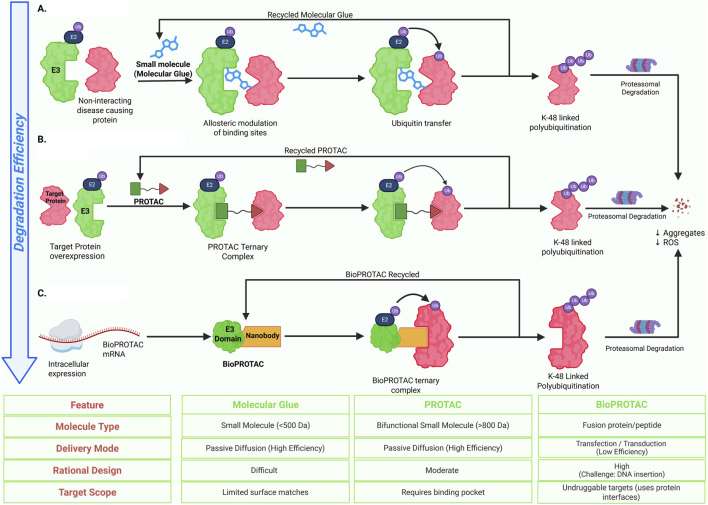
Targeted protein degradation strategies exploiting E3 ubiquitin ligases. **(A)** Molecular glues: Small molecules stabilize or induce interactions between an E3 ligase and a target protein, enabling K48-linked polyubiquitination and proteasomal degradation. **(B)** PROTACs: Bifunctional molecules simultaneously bind an E3 ligase and a target protein, forming a ternary complex that drives catalytic K48-linked polyubiquitination and proteasomal degradation. **(C)** BioPROTACs: Genetically encoded E3–binding fusion proteins recruit endogenous E3 ligases to selected targets, resulting in K48-linked polyubiquitination and proteasomal degradation. Created in BioRender. Krishnakumar, P. (2026) https://BioRender.com/2syn0mm.

The conventional mechanism of small-molecule drug inhibitors is via competitive inhibition. However, the latest framework of inhibitor action, specifically for PICs (proximity-inducing compounds) like PROTACs uses E3 ligase action to induce targeted protein degradation (Churcher, 2018). In spite of the vast library of E3 ligases encoded in the human DNA (more than 600), current PROTAC strategies utilise only a few. This is because of the unavailability of high-affinity, drug-like ligands for the ligases. The most commonly used E3s include VHL, CRBN, IAPs, and MDM2 (Lai and Crews, 2017). Distinct E3 ligase–based targeted protein degradation strategies, including molecular glues, PROTACs, and BioPROTACs, are schematically compared as illustrated in Figures 3A–C, respectively.

PROTACs are heterobifunctional molecules (having two different functional parts). They consist of one ligand for the protein of interest, and another ligand for the E3 ligase that is connected by a chemical linker molecule. This enables even a single PROTAC molecule to promote ubiquitination as well as elimination of multiple target protein molecules. PROTACs disrupt protein complexes, modulate non-enzymatic functions, and can even overcome resistance due to mutations (Bondeson et al., 2015). PROTAC-mediated degradation efficacy is dependent on the E3 ligase interaction and not just the binding affinity. For instance, in VHL-based PROTACs studies, where structure-guided development of PROTACs to target the VHL-HIF1α was performed (Galdeano et al., 2014), the high efficiency in E3 ligase engagement enabled reliable target degradation in both cellular and *in-vivo* conditions. Such studies proved VHL as being a high quality E3 ligase for PROTACs development (Diehl and Ciulli, 2022). CRBN (cereblon) being discovered as a molecular target of thalidomide further expanded the application scope of PROTACs, and hence, structural analyses of CRBN-thalidomide complexes helped improve ligand design. Since then, CRBN-based PROTACs have been shown to be a versatile tool in targeting multiple proteins, including FKBP12, and thereby becoming widely used in targeted protein degradation (Fischer et al., 2014).

Other E3 ligases illustrate both the potential and limitations of ligase selection in PROTAC design. IAP-based degraders (like SNIPERs) can promote degradation of targets via cIAP1-mediated ubiquitination; however, they can also often trigger self-degradation. This limits continued and sustained activity, which causes limited degradation efficiency (Naito et al., 2019). Initial studies of MDM2-based PROTACs also displayed poor degradation efficiency as well as poor physicochemical properties, thereby leading to alternative strategies to improve performance (Li et al., 2019).

A key characteristic of PROTAC-mediated degradation is that it is not solely dependent on binary binding, it is dictated by various factors. Another key factor is the formation of a productive ternary complex between the target protein, the PROTAC molecule as well as the recruited E3 ligase. Studies have shown that a favourable ternary complex cooperativity and geometry is critical to ubiquitination efficacy (Gadd et al., 2017). Thereby, increasing the concentration of PROTAC molecules does not really increase the scale of degradation. This is a “hook-effect”, excessive binary complex formation leads to reduced productive ternary assembly. Hence, it is crucial that PROTAC design is accompanied with careful consideration of E3 ligase and target protein compatibility as well as molecular architecture instead of ligand affinity scores alone (Bondeson et al., 2015; Békés et al., 2022).

There are indications that the specificity of ligase selection may also play an important role in addition to the efficiency of degradation. The ligase selected for a particular PROTAC influences its pharmacokinetic/pharmacodynamic profile, tissue selectivity and clinical tolerance. Through the application of differential E3 ligase expression between tissues and the differentially expressed microenvironment of specific diseases, tissue-selective protein degradation can be accomplished. Transcriptomics and proteomics have shown there is considerable variability in the levels of expression of various E3 ligases. Therefore, through this variability in ligase levels, it may be possible to selectively degrade proteins in disease tissue while reducing systemic toxicity (Nabet et al., 2018). As such, the E3 ligase repertoire will continue to grow, which could promote the discovery of alternative favorable therapeutic strategies. Ultimately, this could lead to the development of adaptable and context-specific PROTAC design (in addition to the VHL and CRBN), thus addressing one of the main limitations to the development of targeted protein degradation (Békés et al., 2022).

### 5.2 Role of E3 ligases in improving CAR T-Cell therapy

CAR T-cell therapy is the genetic engineering of self T-cells to selectively target malignant cells in the body. This has revolutionised the field of cancer therapeutics, and has yielded particularly encouraging results in hematological malignancies (Maude et al., 2014). Despite this, there are persisting challenges like T-cell exhaustion and suppression by tumor microenvironments (TME), which are immunosuppressive in nature; this has led to limited efficacy in solid tumors (Wherry and Kurachi, 2015). Therefore, strategies that reprogram intracellular signaling pathways regulating T-cell activation, metabolic fitness, and inhibitory response expression are required to address these limitations. E3 ligases are crucial regulators in all of these processes that dictate T-cell fate decisions and immune responsiveness. Hence, manipulation of E3 ligases could theoretically be a viable strategy, specifically in the context of solid tumours (Lane et al., 2024; Kumar et al., 2021).

**FIGURE 4:**
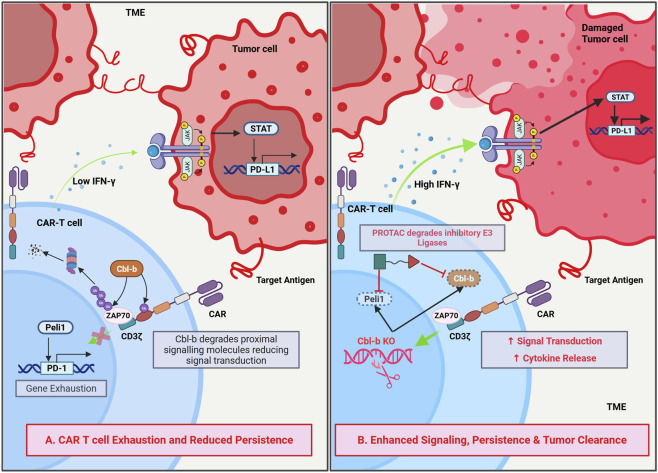
E3 ligase–mediated regulation of CAR T-cell signaling, exhaustion, and persistence in the tumor microenvironment. **(A)** Under immunosuppressive tumor microenvironment (TME) conditions, E3 ligases such as Cbl-b and Peli1 negatively regulate CAR T-cell signaling by promoting ubiquitination and degradation of proximal signaling components, including ZAP70 and CD3ζ. This results in reduced signal transduction, diminished cytokine production, increased PD-1 expression, and functional exhaustion of CAR T cells, ultimately limiting antitumor efficacy. **(B)** Targeted disruption or degradation of inhibitory E3 ligases enhances CAR T-cell signaling strength, cytokine release, and persistence. Reduced E3 ligase–mediated negative feedback restores effector function, increases interferon-γ (IFN-γ) signaling, and improves tumor cell damage and clearance, highlighting E3 ligases as modulators of CAR T-cell efficacy and therapeutic durability. Created in BioRender. Krishnakumar, P. (2026) https://BioRender.com/ws9zf5k.

Several specific E3 ligases have shown to be key players in T-cell exhaustion through modulation of their target negative regulators. One E3 ligase that has received significant attention as being involved in T-cell exhaustion is Cbl-b, which serves as a negative regulator of TCR signaling. The primary mechanism by which Cbl-b restricts T-cell function involves its ability to ubiquitinate molecules early in the cell cascade signaling pathway (Jeon et al., 2004), thereby restricting proliferation and cytokine secretion. Furthermore, Cbl-b promotes the expression of inhibitory receptors including PD-1 and Tim-3 within the TME. To address the detrimental effect of Cbl-b on T-cell function, researchers used CRISPR-Cas9 gene editing to generate Cbl-b deficient cells and tested these cells in preclinical colon cancer models. These studies found that Cbl-b deficient CAR T-cells exhibited superior tumor control and efficacy compared to wild-type CAR T-cells. Importantly, these studies demonstrated that Cbl-b deficient CAR T-cells were able to sustainably produce cytokines and exhibit enhanced cytotoxic activity for longer periods of time (Meng et al., 2018). The role of E3 ligases as modulators of CAR T-cell signaling, exhaustion, and persistence within the tumor microenvironment is schematically illustrated in Figures 4A,B.

CAR T cell function is also negatively impacted by immune checkpoint signaling. FBXO38 is an F-box protein that assembles with other components to form SCF ubiquitin ligase complexes, which target PD-1 for proteasome-mediated degradation thereby reducing inhibitory signals delivered through PD-1 to T cells (Meng et al., 2018). The possibility to engineer CAR T cells modified with FBXO38 has been restricted but it does suggest one potential approach to decrease the impact of PD-L1 mediated immunosuppressive effects on CAR T cells without permanently disrupting the immune checkpoint.

BioPROTACs (biological proteolysis targeting chimeras) represent a novel synthetic biology approach. These engineered bifunctional proteins combine a target, recognition domain with an E3 ligase, binding domain, enabling precise degradation of intracellular targets such as ZAP70, a kinase central to TCR signaling (Kim et al., 2024). Controlled degradation of ZAP70 can reduce overactivation, limit cytokine release syndrome (CRS), and preserve CAR T-cell viability without compromising anti-tumor function. Another target, SMAD2/3, involves using synthetic substrate receptors (SySRs) that recruit E3 ligases to degrade SMAD2/3, transcription factors activated by TGF-β. Given the TME’s high TGF-β levels, SMAD2/3 degradation renders CAR-T cells resistant to immunosuppressive signals. Preclinical studies confirm enhanced CAR T efficacy in solid tumors with elevated TGF-β (Lane et al., 2024).

Overall, these studies demonstrate that E3 ligase engineering is a potential framework for the precise, fine-control type of tuning of CAR T-cell signaling, and could be extremely useful in improving persistence, eliminating exhaustion, and increasing efficacy in solid tumors.

### 5.3 AI–driven therapeutic design in E3 ligase–based modalities

**FIGURE 5:**
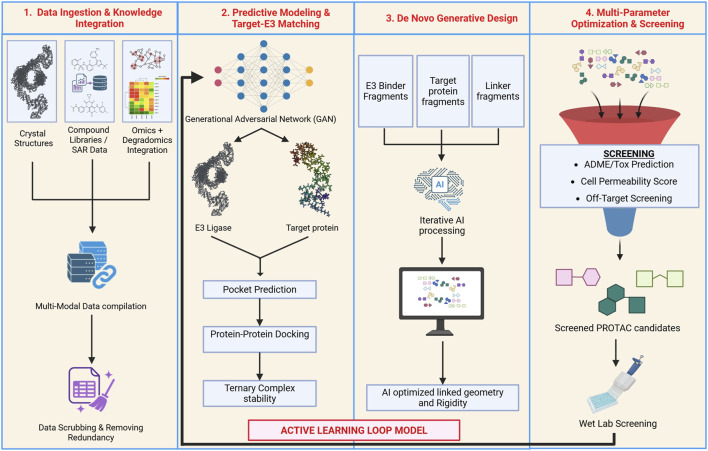
AI-assisted workflows for E3 ligase–based therapeutic design and PROTAC discovery. Schematic representation of an integrated artificial intelligence–enabled pipeline for targeted protein degradation (Dikic, 2017). Multimodal data ingestion combines structural information, compound libraries, structure–activity relationship (SAR) data, and omics/degradomics datasets to construct a curated knowledge base (Finley, 2009). Predictive modeling and target–E3 ligase matching leverage machine learning approaches for interactome mapping, pocket prediction, protein–protein docking, and evaluation of ternary complex stability (Wang et al., 2013). *De novo* generative design integrates E3-binding, target-binding, and linker fragments through iterative AI-driven optimization to refine linker geometry and molecular rigidity (Berndsen and Wolberger, 2014). Multi-parameter optimization and virtual screening prioritize candidate degraders based on predicted pharmacokinetics, permeability, and off-target risk, yielding high-confidence PROTAC candidates for experimental validation. An active learning loop enables continuous refinement of model performance through integration of computational predictions and wet-lab feedback. Created in BioRender. Krishnakumar, P. (2026) https://BioRender.com/prlmg2z.

The increasing application of E3 ligases as therapeutic agents has shown that ubiquitination is regulated through complex, non-linear interactions among E3 ligases, their substrates, and other regulatory factors, as opposed to a linear relationship between an enzyme and its target. Studies using large-scale proteomics and ubiquitinome analyses, provide evidence of how E3 ligases interact with many different substrates under different conditions, with the ability of E3 ligases to recognize substrates being highly dependent on both intrinsic disorder and PTMs, and dynamic protein-protein interactions. Because of the complexity and redundancy of the ubiquitin–proteasome system, several studies have argued that understanding E3 ligase–substrate relationships require systems-level computational analysis rather than characterization of individual interaction pairs (Nabet et al., 2018; Guharoy et al., 2016). An integrated AI-assisted workflow for E3–target interactome mapping, predictive modeling, *de novo* degrader design, and multi-parameter optimization is schematically illustrated in Figure 5 (panels 1–4).

AI analysis is being considered in addition to the use of E3 ligase-based drug treatments to address the major drawback of the limited number of E3 ligases currently used in therapy. A large portion of the most commonly used ligases (such as CRBN and VHL) were discovered as ligands historically, not based on their overall biological relevance or utility. Therefore, researchers have suggested that by using integrative computational models that include structural modeling, tissue-specific gene expression data, and proteomics, researchers can identify potential new E3 ligases for future research and treatment options. These studies define AI as a facilitator layer for ligase identification from a biologically relevant perspective, instead of defining it as a method to predict which therapeutic strategies will be the best. Previous studies describe AI as a unifying design framework for E3 ligase–based therapeutics, enabling systems-level interpretation of ubiquitin signaling networks and rational prioritization of E3 ligases (Békés et al., 2022; Nabet et al., 2018).

## 6 Challenges and future directions

Even though there has been significant progress in areas like PROTACs and engineered CAR T-cells, there are still several challenges that limit clinical integration of E3 ligase-based therapeutics. There are still unresolved gaps with regards to the scope, druggability, specificity, and safety of PROTACs even though they have demonstrated promising results in preclinical and early-clinical activity. The fact that the E3 ligase interactions with substrate and ligands are not completely understood, due to the intrinsic complexity in their mechanisms, is a primary limitation. Much of the progress to date has relied on conventional trial-and-error strategies, which are increasingly limited in the context of combinatorial degrader design, promoting the requirement of AI and ML usage in E3-based therapeutics (Békés et al., 2022).

The fact that there is a very limited usage of the E3 ligase repertoire has led to a very small number of therapeutic directions. This is primarily due to the incomplete knowledge of E3-substrate relationships and limited availability of high-affinity ligands. Along with this, the large molecular size of PROTACs, as well as their mechanistic complexity, makes it difficult to identify more viable systems. They often violate Lipinski’s Rule of Five, and result in poor oral bioavailability, poor solubility, and permeability. Consequently, varying ligase systems present variations in pharmacological profiles (Mullard, 2021).

The advances achieved by AI and ML are now allowing for advancements in both degrader design through prospective methods, and prioritizing the best E3 ligase for use cases for each degrader. Recent reports have included molecular generation strategies in combination with ML-based filtering and structure-guided evaluations for PROTAC design. A notable study uses PROTAC-RL (Zheng et al., 2022), a deep generative reinforcement learning framework for designing BRD4-targeting PROTACs. Thousands of candidate molecules generated by the framework were screened and then refined. The result was the identification of multiple degraders that could induce efficient degradation of the target protein at the cellular level while possessing favorable pharmacokinetic profiles (Zheng et al., 2022).

Deep learning approaches are increasingly being applied to support the discovery of molecular glues, where small and context-dependent protein–protein interactions make rational design challenging. By leveraging structural features, binding energetics, and interaction data, ML models can identify and optimize glue-like molecules that stabilize E3 ligase–substrate complexes. In parallel, AI-accelerated virtual screening frameworks improve discrimination between true binders and decoys across underexplored components of the ubiquitin–proteasome system, enabling scalable identification of high-confidence ligands for E3 ligase–mediated targeted protein degradation (Adeshina et al., 2020; Zhou et al., 2024).

Beyond small-molecule degradation, CAR T-cell–specific challenges introduce additional complexity. Broad E3 substrate spectra raise concerns regarding unintended degradation of essential signaling proteins or inducing systemic toxicity (Guharoy et al., 2016). Safety risks such as cytokine release syndrome and neurotoxicity have motivated the development of inducible safety switches and small-molecule–controlled degradation systems (Klopp et al., 2021). Moreover, heterogeneity of the tumor microenvironment—including hypoxia, metabolic stress, and immune checkpoint signaling—can limit CAR T-cell persistence and efficacy, highlighting the need for context-sensitive E3 ligase circuits (Kosti et al., 2021).

A number of challenges have emerged in recent years that highlight the need for spatially, temporally, and microenvironment-specific regulation of E3 ligase activity. In response, emerging living therapeutic platforms are being explored as next-generation delivery strategies capable of overcoming limitations associated with systemic exposure and off-target effects. Genetically engineered bacteria have been shown to preferentially localize to tumors and remodel the surrounding stroma, thereby enhancing the efficacy of targeted therapies and immunotherapies (Thomas et al., 2022). Building on this concept, engineered probiotic-based systems have enabled epitope-agnostic radionuclide delivery to solid tumors with high localization independent of tumor antigen heterogeneity (Siddiqui et al., 2023). More recently, orally administered engineered microbes have demonstrated utility as both antiviral treatments and vaccines, underscoring the versatility of programmable microbial platforms (Kamble et al., 2025). Notably, the facultative anaerobic growth of these strains enables preferential activity within hypoxic and immunosuppressive microenvironments, providing a rationale for context-specific deployment of E3 ligase–based modalities such as BioPROTACs, molecular glues, and inducible degradation pathways. Collectively, these advances illustrate how innovations in delivery technology are likely to shape the future clinical translation of E3 ligase-based therapeutics.

Future progress in this field is dependent on the extent of expansion of the E3 ligase toolbox. It also would require improvements in ligand discovery techniques (based on AI-assisted virtual screening), as well as structural proteomics being used along with clinical datasets. Advances in delivery technologies—including programmable living therapeutics—will be critical in enabling spatially and temporally controlled deployment of E3 ligase–based interventions (Smith et al., 2017). Developments in all these areas would potentially dictate the design of a “next-generation” PROTACs framework, molecular glues, or CAR T-cell therapeutics. This is crucial in helping develop more precise yet scalable protein degradation strategies.
